# Antitumor Effects of Collagen Peptide Nanoemulsion and Nanoliposome Prepared from Taiwan Tilapia Skin in a Mouse Model

**DOI:** 10.3390/ijms27177835

**Published:** 2026-09-01

**Authors:** Baskaran Stephen Inbaraj, Kuo-Chiang Hsu, Bing-Huei Chen

**Affiliations:** 1 Department of Food Science, Fu Jen Catholic University, New Taipei City 242062, Taiwan; 138547@mail.fju.edu.tw; 2 Department of Nutrition, China Medical University, Taichung 404328, Taiwan; kchsu@mail.cmu.edu.tw

**Keywords:** tilapia skin, collagen peptides, nanopeptides, folic acid-chitosan, lung cancer, animal model

## Abstract

Globally, cancer has emerged as a major cause of human death, and the traditional therapy suffers several drawbacks such as poor survival rate, potential side-effects and increased drug resistance. Recently, natural bioactives obtained by valorization of food and agricultural wastes have drawn great attention as a promising alternative cancer therapeutic. This study aims to evaluate the antitumor efficiency of low-molecular-weight collagen peptides (TSP) isolated from Taiwan tilapia skin, as well as their nanoemulsion (TSPE) and nanoliposome (TSPL) without or with folic acid-chitosan (FC) ligand conjugation (TSPE-FC and TSPL-FC) in comparison with paclitaxel using a nude mice model. Based on the optimized enzymatic hydrolysis conditions, TSP was prepared using alcalase 12.5%, sample-to-water ratio 1:30 (*w*/*v*), pH 8, temperature 50 °C and hydrolysis time 6 h. TSP containing a high proportion of 172 Da fragment (48%) and hydrophobic amino acids (67.2%) was subsequently incorporated into two nanoformulations, with TSPE being prepared with an optimal ratio of soybean oil, lecithin, glycerol, Tween 80 and deionized water, while TSPL was prepared with ethanol, cholesterol, phosphatidylcholine, Tween 80, glycerol and deionized water. The mean particle sizes of TSPE and TSPL were, respectively, 16.1 nm and 33.9 nm, which changed to 15.2 nm and 35.3 nm upon FC conjugation, along with a shift in zeta potential from negative (−41.7 mV for TSPE and −46.6 mV for TSPL) to positive (+23.2 mV for TSPE and +23.9 mV for TSPL), which is favorable for tumor cell penetration and uptake. Nanopeptides showed a sustained release of TSP in an in vitro release study compared to a rapid release from nonencapsulated TSP. Following treatment of A549 cell-induced nude mice tumors with TSP, TSPE, TSPL, TSPE-FC and TSPL-FC at two different doses (100 and 300 mg/kg body weight (BW)) as well as paclitaxel (15 mg/kg BW), the tumor volume, respectively, decreased by 22.4–43.3%, 40.4–70.6%, 36.9–69.2%, 59.1–78.6%, 71.5–85.7% and 90.9%, while the tumor weight reduced by 18.2–48.0%, 42.6–73.0%, 36.1–69.6%, 58.8–79.5%, 69.6–85.2% and 90.3%. In addition, a decrease in the levels of epidermal growth factor (EGF) and vascular endothelial growth factor (VEGF) were shown in mice serum for all treatments, with paclitaxel being the most effective in inhibiting tumor growth, followed by TSPL-FC/TSPE-FC, TSPE/TSPL and TSP. Collectively, both nanoencapsulated peptides and FC conjugated nanopeptides showed the most pronounced effect in inhibiting lung cancer tumors in mice.

## 1. Introduction

Cancer is one of the dominant causes of death globally, with the World Health Organization (WHO) reporting an estimated 20 million new cases and 9.7 million deaths in 2022, and a projected increase to 35 million new cases in 2050 [1,2,3]. Of the various cancer types, lung and breast cancers are the most prevalent ones, respectively, among men and women worldwide, with a lung cancer incidence of 2.5 million and mortality of 1.8 million being reported in 2022 for both men and women [1,2]. In Taiwan, cancer has been the leading cause of death over the past 43 years, with lung cancer ranking first, with an estimated mortality of 54,032 for all cancer types and of 10,495 for lung cancer in 2024 [4,5]. Given the potential side-effects of conventional cancer therapies [6,7,8,9], a more holistic natural approach is necessary, as strong evidence in the reduction of cancer risk has been recently demonstrated through diet control, good living habits and intake of natural health foods/supplements [8,9,10]. However, medical advice from an oncologist is important prior to the intake of such supplements. Thus, it is pivotal to develop natural anticancer agents from fish waste such as tilapia skin with minimal side-effects for effective prevention or treatment of cancer.

Fish is a highly consumed marine food owing to its high protein content and healthy lipid composition, vitamins and minerals [11]. It was reported that 20.2 kg per capita of fish was consumed globally in 2020, with an estimated rise to 21.4 kg per capita by 2030 [12]. Consequently, a substantial amount of byproducts, representing 50–70% of total fish weight, can be generated as waste with a significant amount of recoverable protein from trimmings (12–22%), spleen and fish roe (14–27%), gut and viscera (9–23%), bones (10–15%), head (11–13%) and skin (8–12%) [6,13,14]. Production of fish protein hydrolysates and bioactive peptides (2–20 amino acids (AA)) by chemical or enzymatic means of fish waste-derived protein has been shown to be an effective strategy to obtain functional products through valorization of fish waste byproducts [6,15,16,17]. However, the chemical hydrolysis method is unsuitable for food applications because of food safety issues. Instead, enzymatic hydrolysis offers a green production of low-molecular-weight (MW) bioactive peptides rich in hydrophobic AA with tailored functional properties through selective cleavage of specific peptide bonds depending on the enzyme variety and its concentration, hydrolysis time, temperature and pH [18,19,20,21]. Such fish protein hydrolysates have been reported to exert various biological activities including antioxidant, antimicrobial, anticancer, antidiabetic, wound healing, antihypertensive and antiosteoarthritis effects [13,15,22,23,24,25]. Thus, fish waste-derived bioactive peptides obtained by enzymatic hydrolysis can be a promising therapy for cancer treatment. However, undesirable off-flavor, poor absorption in vivo, hygroscopic nature and instability under gastrointestinal conditions limits their applications in the food and drug industry [18,26].

Nanoencapsulation of bioactive compounds has been shown to effectively improve their stability and solubility for enhanced absorption, cell membrane permeability and bioactivity with controlled release and minimal toxicity [13,24,26]. Owing to the large surface-area-to-volume ratio, surface modification capability and prolonged blood circulation, various colloidal nanodelivery systems including nanoliposomes, microemulsion and nanoemulsion have been explored for encapsulation of drugs and functional compounds, including bioactive peptides [18]. As they do not possess tumor specificity, both passive and active targeting of drugs/bioactive compounds towards tumor sites can be achieved through enhanced permeability and retention (EPR) effects for the former, and by conjugating with tumor specific ligands for enhanced cellular uptake for the latter [8]. Folic acid is particularly effective as a tumor-targeting ligand in binding to folate receptors overexpressed on the surface of most cancer cells including lung, ovarian, breast, kidney, colon, bladder, brain and cervical [27,28]. Likewise, chitosan, being highly stable, nontoxic and rich in amino and OH groups, exhibits prolonged blood circulation and increased solubility in the acidic tumor environment for improvement of transport and sustained release of bioactive compounds [8,29]. Therefore, the preparation of bioactive peptide nanoemulsion or nanoliposome with subsequent conjugation to a folic acid-chitosan (FC) ligand can be a promising approach for improving their anticancer efficiency by active targeting with minimal side effects.

Given its fast-growing speed and wide adaptability, tilapia (*Oreochromis niloticus*) is extensively farmed, with a worldwide production of 4.2 million tons reported in 2016 [30]. Taiwan is the second largest producer of tilapia fish (71,030 tons/year), exporting to both USA and Japan [31]. As tilapia skin contain 28–40% collagen (dry weight), it can be used as a potential raw material for producing hydrolysates rich in bioactive peptides [32]. In our previous study, collagen peptides (TSP) containing a high proportion of peptide fragment at 172 Da was produced by enzymatic hydrolysis of acid-soluble collagen (TSC) from Taiwan tilapia skin using 12.5% alcalase [24]. Following encapsulation into nanoemulsion, nanoliposome and nanogold without and with FC ligand conjugation, nanoliposome-FC was demonstrated to possess a much higher inhibition effect against human lung cancer cells A549, followed by nanoemulsion-FC, nanoemulsion, nanoliposome, nanogold-FC, nanogold and peptide, with cell cycle arrest at G2/M phase and a rise in the proportion of necrotic/late apoptotic cells, as well as an increase in caspase-3, caspase-8 and caspase-9 activities for apoptosis execution [24]. However, the antitumor efficiency of nanopeptides using an animal model still remains unexplored. Therefore, in this study, we aim to evaluate the antitumor efficiency of low-MW collagen peptides isolated from Taiwan tilapia skin, as well as their nanoemulsion and nanoliposome without or with FC ligand conjugation in comparison with paclitaxel using a nude mice model.

## 2. Results and Discussion

### 2.1. Production and Characterization of TSC and TSP

**Figure 1:**
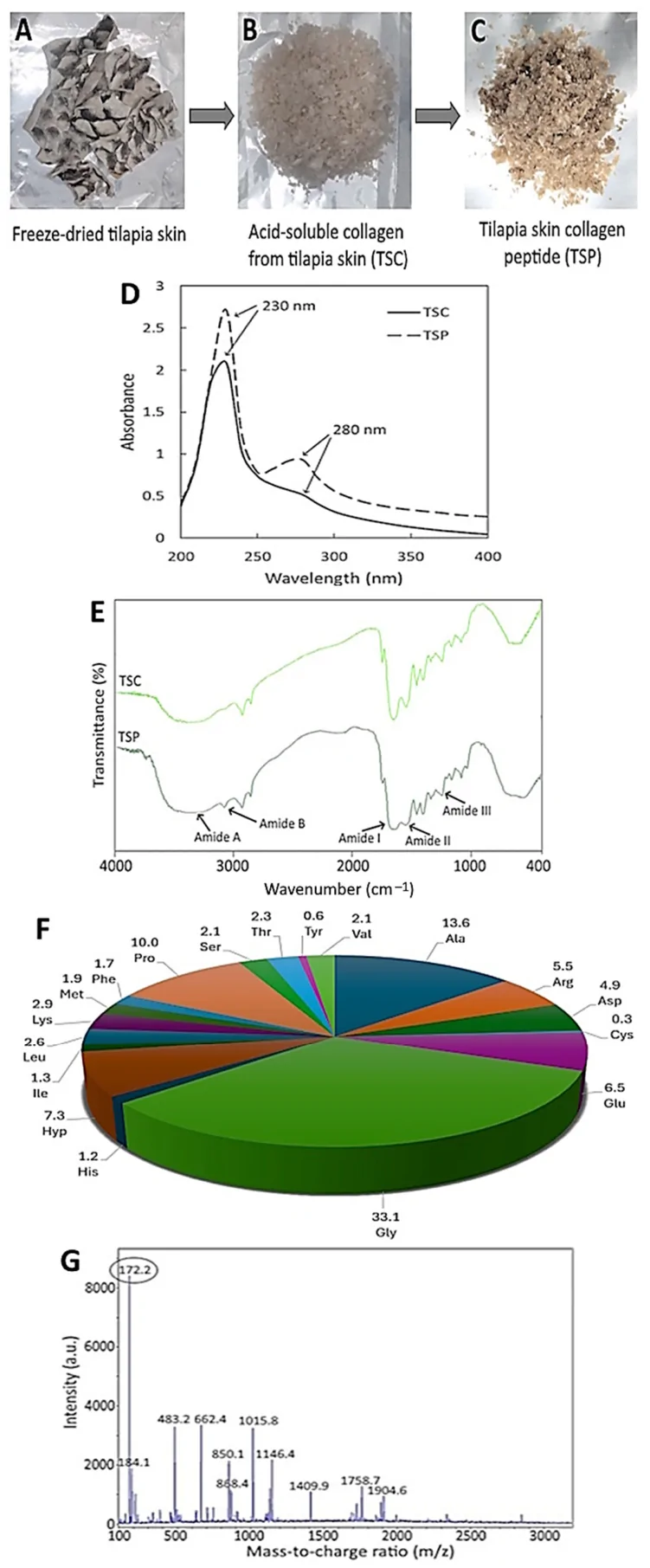
Photographs showing the appearance of freeze-dried tilapia skin (**A**) as well as TSC (**B**) and TSP (**C**) prepared from tilapia skin, along with their characterization by absorption spectra (**D**), FTIR spectra (**E**), AA composition in percentage by HPLC-DAD (**F**) and MW distribution by MALDI-TOF-MS (**G**). Acid-soluble collagen (TSC) was extracted by treating freeze-dried tilapia skin with 0.5 M acetic acid (1:15, *w*/*v*) followed by precipitation with NaCl (2.6 M) in the presence of 0.05 M Tris-HCl (pH 7.5) and dialysis against acetic acid (0.1 M) and water. Collagen peptide (TSP) was prepared by enzymatic hydrolysis with alcalase at 12.5%, pH at 8, temperature at 50 °C and hydrolysis time at 6 h. Analysis of amino acid composition by HPLC-DAD was done in triplicate (*n* = 3), and the mean values are reported in Figure 1F. TSC, tilapia skin acid-soluble collagen; TSP, tilapia skin collagen peptide with MW 172 Da obtained by enzymatic hydrolysis of TSC using 12.5% alcalase; MW, molecular weight; FTIR, Fourier transform infrared spectroscopy; HPLC-DAD, high performance liquid chromatography-diode array detector; MALDI-TOF-MS, matrix-assisted laser desorption/ionization-time of light-mass spectrometry; Ala, alanine; Arg, arginine; Asp, aspartic acid; Cys, cysteine; Glu, glutamic acid; Gly, glycine; His, histidine; Hyp, hydroxyproline; Ile, isoleucine; Leu, leucine; Lys, lysine; Met, methionine; Phe, phenylalanine; Pro, proline, Ser, serine; Thr, threonine; Tyr, tyrosine; Val, valine. TSC, tilapia skin acid-soluble collagen; TSP, tilapia skin collagen peptide.

Figure 1A–C shows the appearance of freeze-dried tilapia skin waste (A) as well as acid-soluble collagen (B) and collagen peptide (C) produced from tilapia skin. The TSC was produced from freeze-dried tilapia skin waste with an extraction yield of 23.36% (Figure 1A,B) by adopting the method described in the Supplementary Materials. Based on an optimized enzymatic hydrolysis condition mentioned in Section 3.1, TSP was prepared by subjecting the TSC isolated from tilapia skin to enzymatic hydrolysis, with the degree of hydrolysis being 16.72% (Figure 1C). During enzymatic hydrolysis, the enzymes generate peptide fragments with low-MW by breaking peptide bonds through disrupting hydrophobic interaction, hydrogen bond and other chemical bonds that stabilize protein secondary and tertiary structures [19]. Specifically, alcalase is an endopeptidase with a broad substrate specificity capable of hydrolyzing most peptide bonds in proteins to yield bioactive peptides with improved solubility and functional properties [11]. By using alcalase, the degree of hydrolysis can be easily controlled to enable production of bioactive peptides rich in hydrophobic AA (key determinant for anticancer activity) with tailored functionalities [20,21].

The presence of two α bands (100–130 kDa) and one β band (250 kDa) in the sodium dodecyl sulfate-polyacrylamide gel electrophoresis (SDS-PAGE) pattern for TSC signified type I collagen (Figure S1A), while the bands for TSP appeared at MW < 3 kDa when compared to that obtained for a standard protein marker (3–250 kDa) (Figure S1B). Two absorption peaks for peptide bond (230 nm) and aromatic AA (280 nm) (Figure 1D) and typical Fourier transform infrared spectroscopy (FTIR) amide I, II and III bands (around 1659, 1552 and 1241 cm^−1^), along with amide A and B bands (around 3308 and 2928 cm^−1^) (Figure 1E), further confirmed that TSC is type I collagen. The AA analysis of TSC and TSP as determined by high-performance liquid chromatography-diode array detection (HPLC-DAD) showed the presence of 18 AA, with the content ranging from 0.26 to 32.8% and 0.30 to 33.1%, respectively (Figure 1F; Table S1). MW distribution analysis of TSP by matrix-assisted laser desorption/ionization time-of-flight mass spectrometry (MALDI-TOF-MS) showed a total of 11 fragments in the *m*/*z* range of 172–1905 Da, with a dominant low-MW peptide fragment at *m*/*z* 172 Da contributing to 48% of the total fragment proportion (Figure 1G). Zeta potential analysis revealed a positive surface charge for both TSC and TSP, with values equaling +22.1 and +22.7 mV, respectively (Figure S1C,D). Surface morphology by scanning electron microscopy (SEM) depicted a complex multilayer and fibrillar structure for TSC (Figure S1E), while a sheet-like structure with folded and entangled surface having sharp edges was observed for TSP (Figure S1F). Both TSP and TSC showed similar absorption and FTIR peaks (Figure 1D,E), along with only a slight variation in zeta potential (Figure S1C,D) and AA composition (Figure 1F), implying that the structural integrity, surface charge and AA composition remained unaffected by enzymatic hydrolysis during TSP preparation [24].

### 2.2. Characterization of TSPE and TSPL Prepared Using TSP

**Figure 2:**
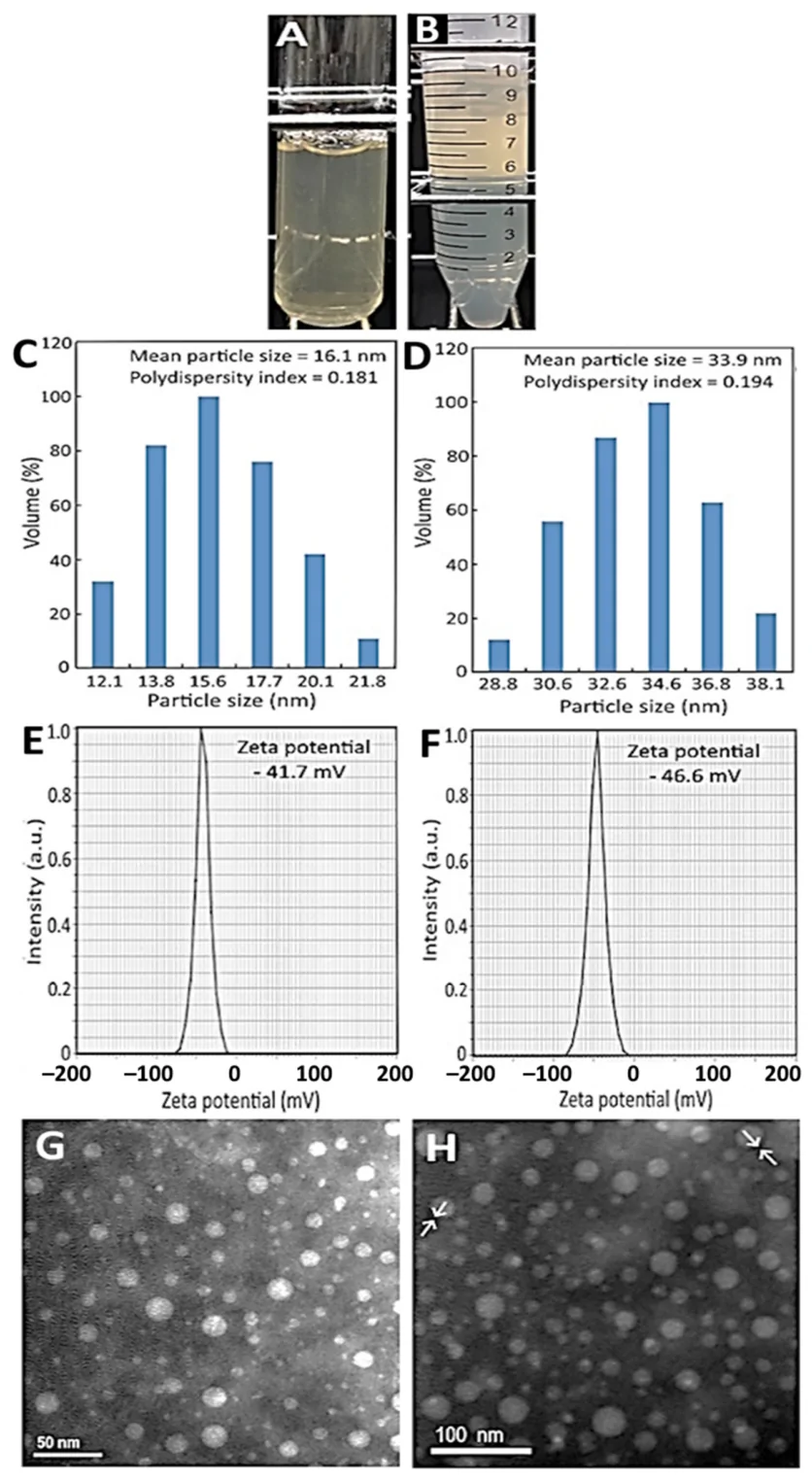
Characterization of nanoemulsion (**A**,**C**,**E**,**G**) and nanoliposome (**B**,**D**,**F**,**H**) prepared from tilapia skin collagen peptide (TSPE and TSPL) showing their appearance (**A**,**B**), particle size distribution based on DLS measurement (**C**,**D**), zeta potential (**E**,**F**) and TEM image (**G**,**H**). Analysis of particle size by DLS and zeta potential were done in triplicate (*n* = 3), and the mean values are reported in (**C**–**F**). The two arrows pointing to each other in (**H**) show the typical lipid bilayer of nanoliposome. TSPE, tilapia skin collagen peptide nanoemulsion; TSPL, tilapia skin collagen peptide nanoliposome; DLS, dynamic light scattering; TEM, transmission electron microscopy.

According to the preparation methods described in Section 2.2, both TSPE and TSPL were successfully prepared for subsequent characterization. Figure 2A–H shows the appearance (A,B), particle size distribution, average particle size and polydispersity index (PDI) (C,D), zeta potential (E,F) and transmission electron microscopy (TEM) image (G,H) of TSPE (A,C,E,G) and TSPL (B,D,F,H). Both TSPE and TSPL appeared transparent and pale-yellow in color (Figure 2A,B), while the particle size analysis by a dynamic light scattering (DLS) instrument revealed an average particle size of 16.1 nm and PDI of 0.181 for TSPE, as well as 33.9 nm and 0.194 for TSPL (Figure 2C,D), implying that nano-sized TSPE and TSPL with homogeneous and narrow size distribution were obtained. A zeta potential with a high-negative value of −41.7 mV for TSPE (Figure 2E) and −46.6 mV for TSPL (Figure 2F) demonstrated that highly stable nanodispersions were produced. Obviously, zeta potential can be used to measure the electrostatic repulsion between the same charged particles, and thus the higher the zeta potential values (either positive or negative), the stronger the repulsion between particles, thereby preventing their aggregation for enhanced stability. On the contrary, a low zeta potential value indicates the existence of weak repulsive forces between particles, causing them to aggregate by van der Waals forces and resulting in the formation of an unstable dispersion. Moreover, a nanodispersion satisfying a zeta potential value of >30 mV or <−30 mV is reported to be highly stable [17]. High-resolution TEM images showed that both TSPE and TSPL were in spherical shape with an apparent lipid bilayer (indicated by arrows), typical of nanoliposomes for the latter (Figure 2G,H). As the phospholipids used to prepare nanoliposomes are amphipathic in nature (containing both hydrophilic phosphate and two hydrophobic fatty acids), they tend to arrange in the form of bilayers in water, with the hydrophilic head facing the water environment (both outside and inside) and hydrophobic tails shielded from water within the bilayer to form a stable structure. Furthermore, the nanoparticle size obtained directly from TEM images (10.0–20.9 nm for TSPE and 21.8–42.3 nm for TSPL) is consistent with that obtained by DLS (Figure 2G,H). The encapsulation efficiency for TSPE and TSPL was determined to be 93% and 84%, respectively.

### 2.3. Characterization of Folic Acid-Chitosan Ligand Conjugated TSPE and TSPL (TSPE-FC and TSPL-FC)

**Figure 3:**
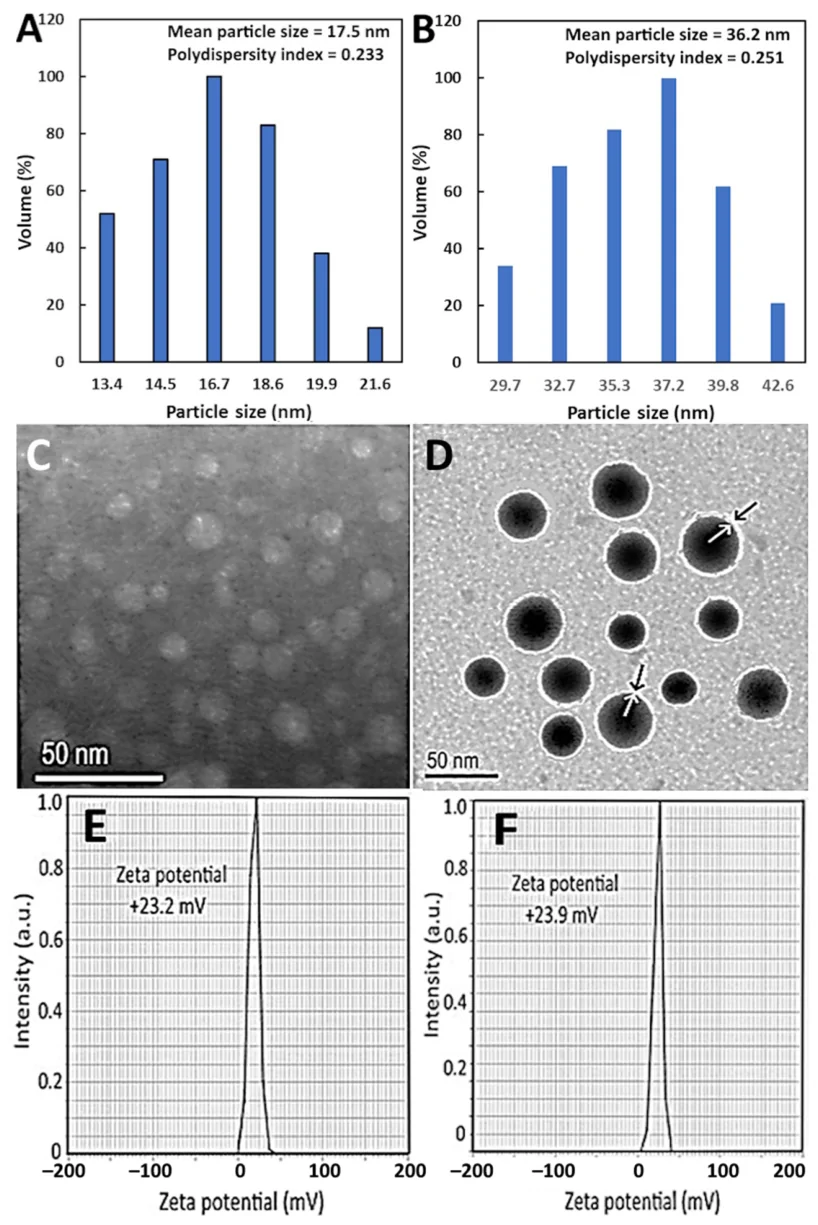
Characterization of folic acid-chitosan ligand conjugated nanoemulsion (**A**,**C**,**E**) and nanoliposome (**B**,**D**,**F**) prepared from tilapia skin collagen peptide (TSPE-FC and TSPL-FC) showing their particle size distribution based on DLS measurement (**A**,**B**), TEM images (**C**,**D**) and zeta potential (**E**,**F**). Analysis of particle size by DLS and zeta potential were done in triplicate (*n* = 3), and the mean values are reported, respectively, in Figure 3A,B and Figure 3E,F. The two white arrows pointing to each other in (**D**) show the typical lipid bilayer of nanoliposome. TSPE-FC, folic acid-chitosan conjugated tilapia skin collagen peptide nanoemulsion; TSPL-FC, folic acid-chitosan conjugated tilapia skin collagen peptide nanoliposome; DLS, dynamic light scattering; TEM, transmission electron microscopy.

To enhance the targeting efficiency of TSPE and TSPL, FC was conjugated with TSPE and TSPL based on the preparation method described in Section 2.3 to obtain TSPE-FC and TSPL-FC. Figure 3A–F shows the characterization of TSPE-FC (A,C,E) and TSPL-FC (B,D,F) for particle size distribution by DLS (A,B), TEM images (C,D) and zeta potential (E,F). Upon conjugation with FC ligand, the pale-yellow TSPE and TSPL were turned to deep-yellow in TSPE-FC and TSPL-FC due to the prominent yellow color of folic acid solution. The average particle size and PDI as determined by DLS were 17.5 nm and 0.233, respectively, for TSPE-FC, as well as 36.2 nm and 0.251 for TSPL-FC (Figure 3A,B), which were all higher than that obtained for TSPE (16.1 nm and 0.181) and TSPL (33.9 nm and 0.194) prior to FC conjugation (Figure 2C,D), implying a successful conjugation of FC with TSPE and TSPL.

It is evident from the high-resolution TEM images that both TSPE-FC and TSPL-FC were spherical in shape (Figure 3C,D), with a visible lipid bilayer (shown by arrows) for the latter (Figure 3D), implying that the conjugation of FC with TSPE and TSPL did not affect their shape (Figure 2G,H and Figure 3C,D), as well as the typical nanoliposome bilayer in TSPL (Figure 2H and Figure 3D). Although TSPL-FC particles are expected to appear white in a dark background due to negative staining by phosphotungstic acid during TEM sample preparation, a reverse trend is shown in Figure 3D, which should be due to a strong interaction of the staining agent with FC ligand conjugated to the surface of TSPL-FC.

Interestingly, zeta potential analysis showed a change from negative to positive value after conjugation of FC with TSPE and TSPL for both TSPE-FC (−41.7 mV to +23.2 mV) (Figure 2E and Figure 3E) and TSPL-FC (−46.6 mV to +23.9 mV) (Figure 2F and Figure 3F), which should be beneficial for their enhanced cellular uptake via interaction with the negatively charged surface of lung tumor A549 cells. However, the zeta potential of both TSPE-FC and TSPL-FC fail to meet the stability range (<−30 mV or >+30 mV) mentioned above [17]. It is worth pointing out that the stability provided by FC ligand due to the steric effect is not taken into account during zeta potential analysis, as the zeta potential analyzer usually measures only the electrostatic stability, but not the steric stability of polymers like chitosan. Based on this phenomenon, both TSPE-FC and TSPL-FC prepared in this study should be quite stable [24,33].

### 2.4. In Vitro Release Kinetics

**Figure 4:**
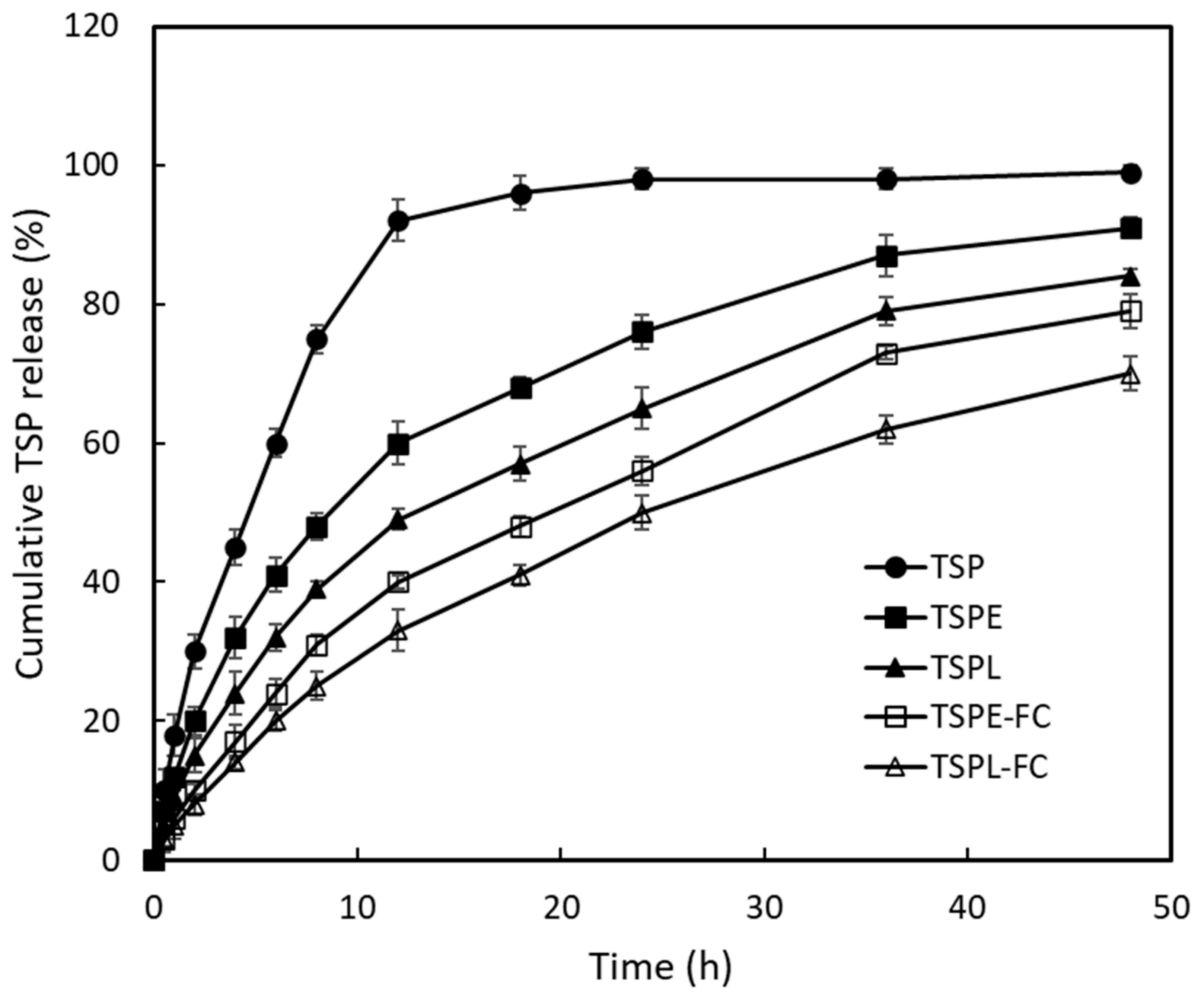
In vitro release kinetics of TSP and TSP encapsulated nanopeptides performed by dynamic dialysis method using 12 kDa MW cutoff dialysis membrane at 37 °C for 48 h. The cumulative release percentage is plotted as the mean ± standard deviation (*n* = 3). TSP, tilapia skin collagen peptide; TSPE, tilapia skin collagen peptide nanoemulsion; TSPL, tilapia skin collagen peptide nanoliposome; TSPE-FC, folic acid-chitosan conjugated TSPE; TSPL-FC, folic acid-chitosan conjugated TSPL.

Determination of in vitro release profile as a function of time is pivotal for evaluating the rate of drug/bioactive compound release and forms a basis for subsequent in vivo studies. Figure 4 shows the in vitro release kinetics of free peptide (TSP) and TSP encapsulated nanopeptides without or with FC conjugation over a period of 48 h. Compared to TSP encapsulated nanoemulsion (TSPE), nanoliposome (TSPL) and their FC conjugated forms (TSPE-FC and TSPL-FC), free TSP showed a rapid release through the dialysis membrane, attaining 92% within 12 h. However, TSPE, TSPL, TSPE-FC and TSPL-FC showed a sustained release, with cumulative percentage at 24 h and 48 h, respectively, being 76% and 91% for TSPE, 65% and 84% for TSPL, 56% and 79% for TSPE-FC and 50% and 70% for TSPL-FC, implying that encapsulation of TSP into a nanosystem could significantly control the release of TSP. Among nanopeptides, TSPL showed a slower release of TSP compared to TSPE, which may be attributed to the protective effect of lamellar lipid bilayer in the former. Likewise, FC conjugated TSPE (TSPE-FC) and TSPL (TSPL-FC) showed a slower TSP release when compared to their non-conjugated forms (TSPE and TSPL), probably caused by the delayed diffusion of TSP through FC coating. Thus, the impact of sustained release among free TSP and TSP encapsulated nanopeptides followed the order: TSPL-FC > TSPE-FC > TSPL > TSPE > TSP. Such sustained release observed for nanopeptides prepared in this study when compared to free peptide revealed that these nanopeptides possess a great potential as carriers for providing stability and sustained delivery of peptides with enhanced biological activity. Several studies have also demonstrated a similar in vitro release behavior, with Sharma et al. [34] reporting 95% and 59% release, respectively, after 24 h from free platein peptide and platein encapsulated nanoliposome prepared from salmon frame, while Hosseini et al. [35] showed a sustained peptide release (41%) from nanoliposome encapsulated rainbow trout fish skin gelatin hydrolysate after 24 h when compared to 99% release after 8 h from its nonencapsulated form.

### 2.5. Antitumor Efficiency

Both peptide and nanopeptide treatments at a low dose at 100 mg/kg and a high dose at 300 mg/kg were chosen initially based on several published articles [36,37,38] and a preliminary study conducted in our lab demonstrating significant biological response/tumor inhibition efficiency and repeat-dose tolerability. For instance, Woo et al. [36] reported a dose-dependent anti-obesity effect in mice by administering skate fish skin-derived collagen peptide at 100, 200 and 300 mg/kg doses through regulation of lipid metabolism. Likewise, marine-derived protein hydrolysates were shown to exert significant antitumor activity in sarcoma tumor-bearing mice at comparable doses, with oyster hydrolysate administered at 200 and 500 mg/kg demonstrating dose-dependent tumor inhibition in mice [37], while tuna protein hydrolysate could exhibit significant tumor inhibition effects at 200 and 400 mg/kg doses [38]. Therefore, three doses each of peptide (TSP), nanopeptides (TSPE and TSPL) and FC conjugated nanopeptides (TSPE-FC and TSPL-FC) at 100, 200 and 300 mg/kg were selected as low, medium and high doses for the preliminary study. Additionally, a positive control drug, paclitaxel, was selected at a dose of 15 mg/kg based on recent published reports [39,40].

Specifically, the preliminary study was conducted in our lab with a total of 51 nude mice by dividing into 17 groups with each group containing 3 mice each (*n* = 3) and administering control (0.85% saline), paclitaxel (15 mg/kg) as well as TSP, TSPE, TSPL, TSPE-FC and TSPL-FC separately at three different doses (100, 200 and 300 mg/kg). The details of different groups, their treatments and doses are described elaborately in the Supplementary Materials. Results showed that, compared to control (without treatment), paclitaxel showed the highest inhibition effect in reduction in tumor volume and weight, followed by TSPL-FC, TSPE-FC, TSPE, TSPL and TSP (Figures S2 and S3). It is evident from Figures S2 and S3 that among three different doses, an insignificant difference (*p* > 0.05) in tumor volume and weight was shown between low-dose and medium-dose treatments, while a significant difference (*p* < 0.05) was shown between the low-dose and high-dose treatments, implying that a low dose of 100 mg/kg and a high dose of 300 mg/kg can be selected as the two optimal doses for TSP, TSPE, TSPL, TSPE-FC and TSPL-FC treatments in the subsequent study. Similarly, given the sustained release observed for all nanopeptides in the in vitro release study shown above, instead of daily administration, the time of treatment dose was chosen as once every two days for low and high doses of TSP, TSPE and TSPL and low doses of TSPE-FC and TSPL-FC, as well as twice every two days (once in the morning and evening) for high doses of TSPE-FC and TSPL-FC. The details of injection volume of each mouse are specified in Section 3.6.1.

**Figure 5:**
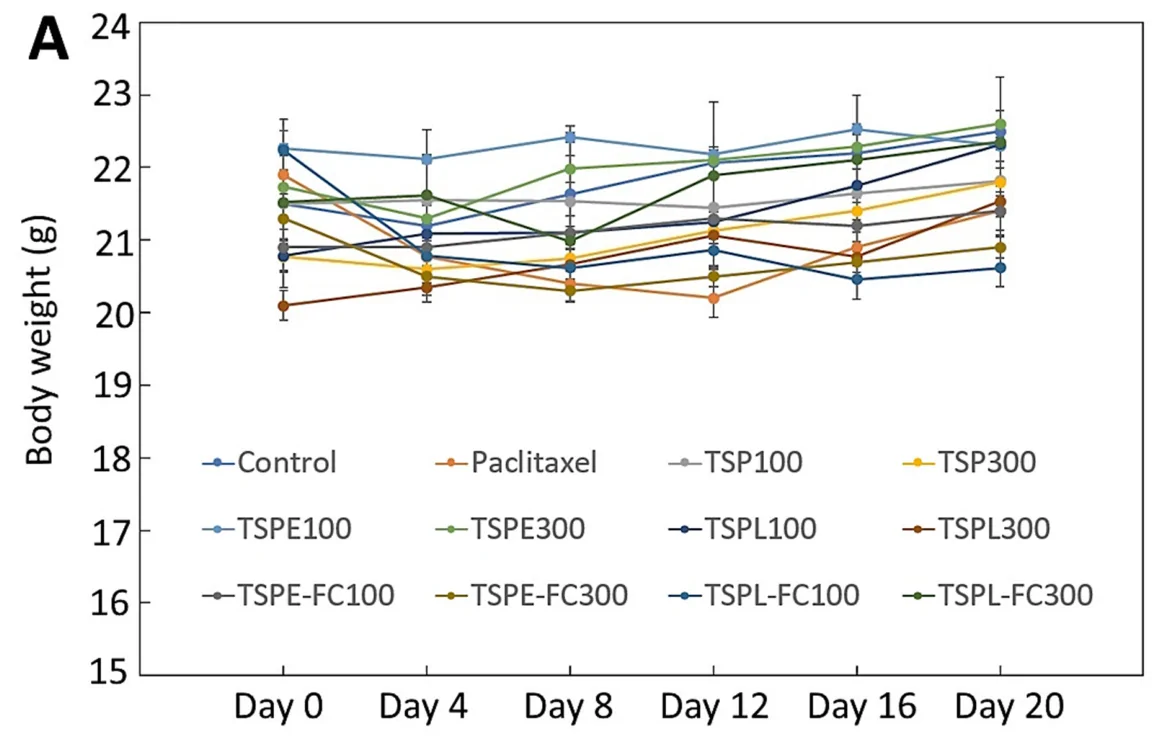

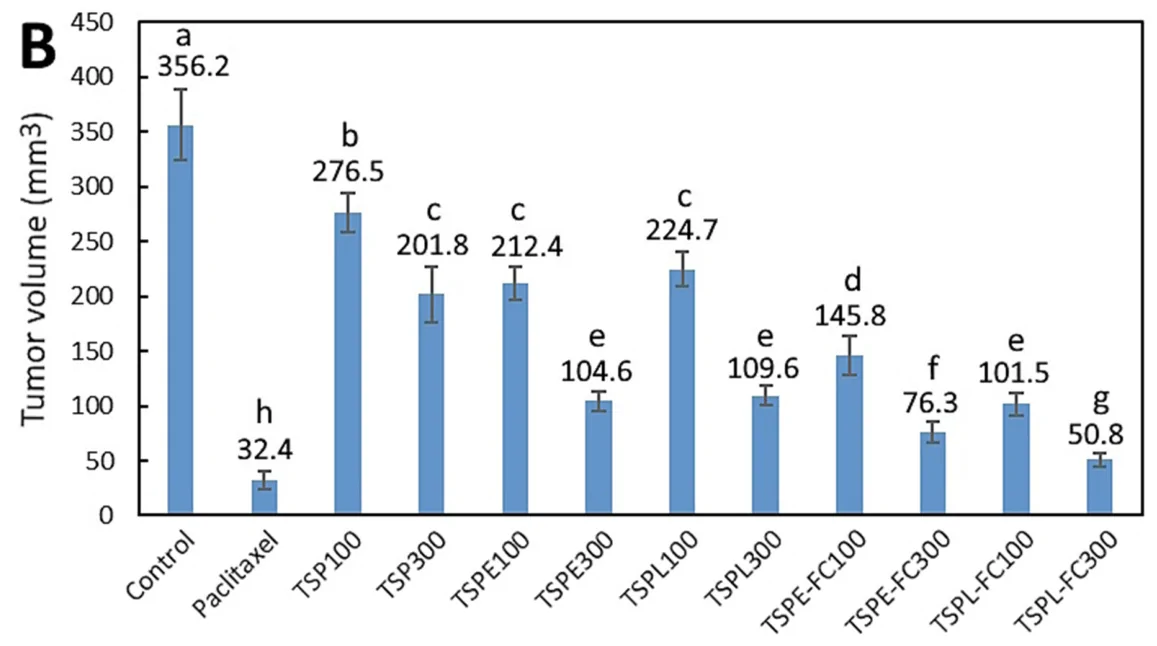
Effect of tilapia skin collagen peptides (TSP) and their nanopeptides (TSPE and TSPL) as well as folic acid-chitosan ligand conjugated nanopeptides (TSPE-FC and TSPL-FC) on body weight (**A**) and tumor size (**B**) of nude mice during the experimental period of 3 weeks. TSP, TSPE, TSPL, TSPE-FC and TSPL-FC each at two different doses (100 and 300 mg/kg BW), paclitaxel at 15 mg/kg BW) and control (0.85% saline) were injected separately for a 3-week treatment period. Data are presented as mean ± standard deviation of measurements from six nude mice (*n* = 6), and the data in Figure 5B with different small letters (a–h) are significantly different (*p* < 0.05). BW, body weight of nude mice.

Compared to the control (without treatment), an insignificant (*p* > 0.05) or minor variation in mice weight was observed following a 3-week treatment period for all eleven treatments (Figure 5A), implying that no marked treatment-associated body-weight loss was observed. Figure 5B shows the volume of mice tumor as affected by different treatments, with paclitaxel being the most effective in reducing the tumor volume by 90.9%, followed by TSPL-FC at 300 mg/kg BW (85.7%), TSPE-FC at 300 mg/kg BW (78.6%), TSPL-FC at 100 mg/kg BW (71.5%), TSPE at 300 mg/kg BW (70.6%), TSPL at 300 mg/kg BW (69.2%), TSPE-FC at 100 mg/kg BW (59.1%), TSP at 300 mg/kg BW (43.3%), TSPE at 100 mg/kg BW (40.4%), TSPL at 100 mg/kg BW (36.9%) and TSP at 100 mg/kg BW (22.4%). However, the reduction in tumor volume was insignificant (*p* > 0.05) among TSPE (300 mg/kg BW), TSPL (300 mg/kg BW) and TSPL-FC (100 mg/kg BW), as well as among TSP (300 mg/kg BW), TSPE (100 mg/kg BW) and TSPL (100 mg/kg BW).

Furthermore, a decrease in tumor volume was observed in a dose-dependent manner for all of the treatments, with nanopeptides (TSPE, TSPL, TSPE-FC and TSPL-FC) being more efficient than peptides (TSP). Additionally, the percentage of tumor volume reduction was from 40.4% to 85.7% for nanopeptides when compared to only 22.4% to 43.3% for peptides. Among the various nanopeptides, FC conjugated nanopeptides TSPE-FC and TSPL-FC were more effective in reducing the tumor volume by 59.1–85.7% than nanopeptides without FC conjugation (40.4–70.6% for TSPE and TSPL). However, at the same nanopeptide dose (100 or 300 mg/kg BW), no significant change (*p* > 0.05) in tumor volume was shown between TSPE and TSPL. However, following a rise in dose from 100 to 300 mg/kg BW, a significant decline (*p* < 0.05) in tumor volume was observed, with a percentage climbing from 59.1% to 78.6% for TSPE-FC and from 71.5% to 85.7% for TSPL-FC.

**Figure 6:**
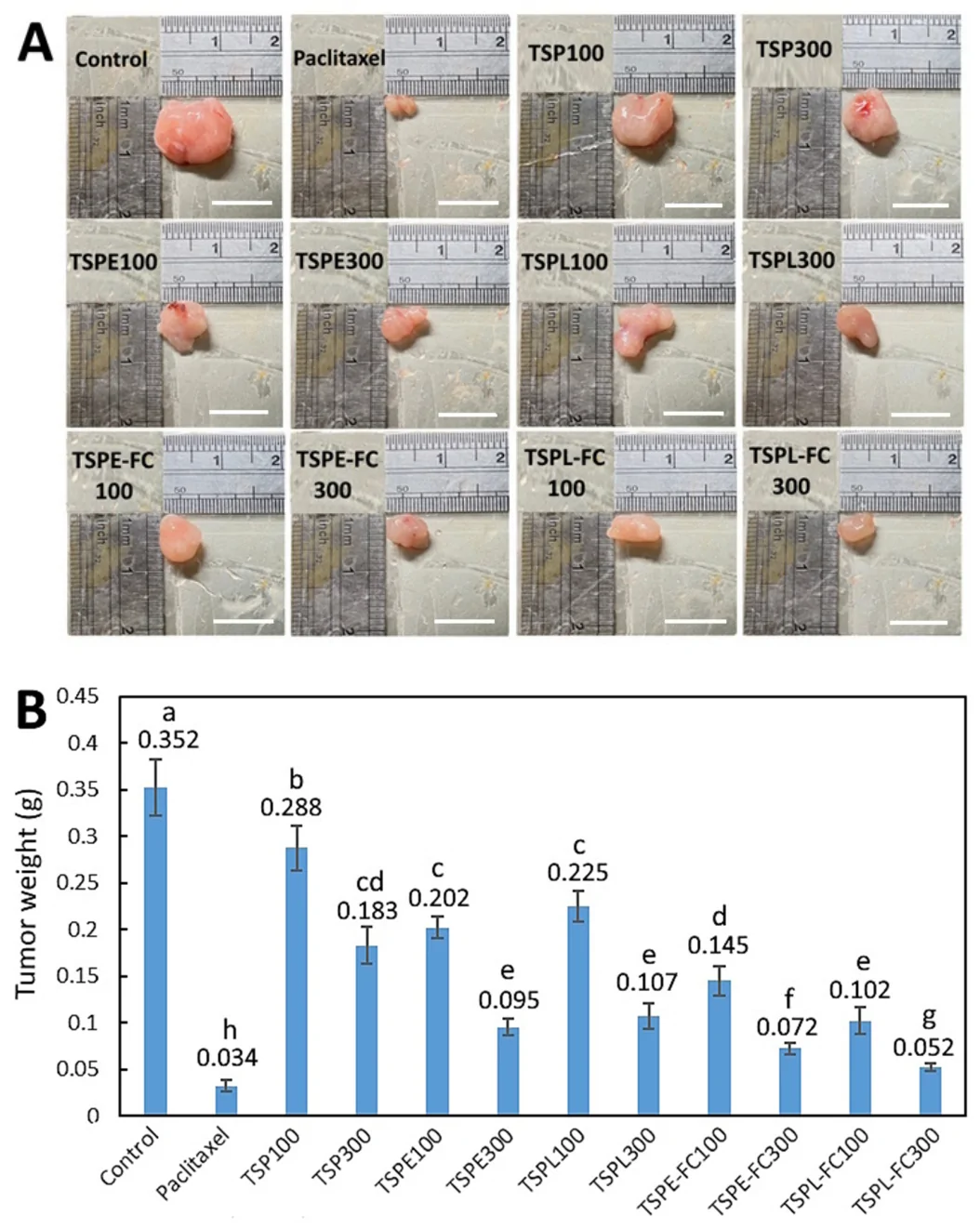
Effect of tilapia skin collagen peptides (TSP) and their nanopeptides (TSPE and TSPL) as well as folic acid-chitosan ligand conjugated nanopeptides (TSPE-FC and TSPL-FC) on visual tumor size (**A**) and tumor weight (**B**) of nude mice after a 3-week treatment period. TSP, TSPE, TSPL, TSPE-FC and TSPL-FC each at two different doses (100 and 300 mg/kg BW), paclitaxel at 15 mg/kg BW) and control (0.85% saline) were injected separately for a 3-week treatment period. Data in Figure 6B are presented as mean ± standard deviation of measurements from six nude mice (*n* = 6), and the data with different small letters (a–h) are significantly different (*p* < 0.05). The scale bars in Figure 6A represent 1 cm. BW, body weight of nude mice.

Figure 6A depicts the photographs of tumors collected from sacrificed mice after a 3-week treatment period, while Figure 6B depicts the effect of different treatments on tumor weight. Both tumor weight and size showed the same trend as tumor volume for all of the treatment groups. Compared to the control, the tumor weight was decreased by 90.3%, 85.2%, 79.5%, 73.0%, 71.0%, 69.6%, 58.8%, 48.0%, 42.6%, 36.1% and 18.2% for paclitaxel, TSPL-FC at 300 mg/kg BW, TSPE-FC at 300 mg/kg BW, TSPE at 300 mg/kg BW, TSPL-FC at 100 mg/kg BW, TSPL at 300 mg/kg BW, TSPE-FC at 100 mg/kg BW, TSP at 300 mg/kg BW, TSPE at 100 mg/kg BW, TSPL at 100 mg/kg BW and TSP at 100 mg/kg BW, respectively (Figure 6B). Similar to tumor volume, a decline in tumor weight and size was observed in a dose-dependent manner for peptides (TSP), nanopeptides (TSPE and TSPL) and FC conjugated nanopeptides (TSPE-FC and TSPL-FC), with paclitaxel (15 mg/kg BW) as well as TSPL-FC, TSPE-FC, TSPE and TSPL treatments at high dose (300 mg/kg BW) being the most efficient.

### 2.6. Regulation of EGF and VEGF in Mice Serum

**Figure 7:**
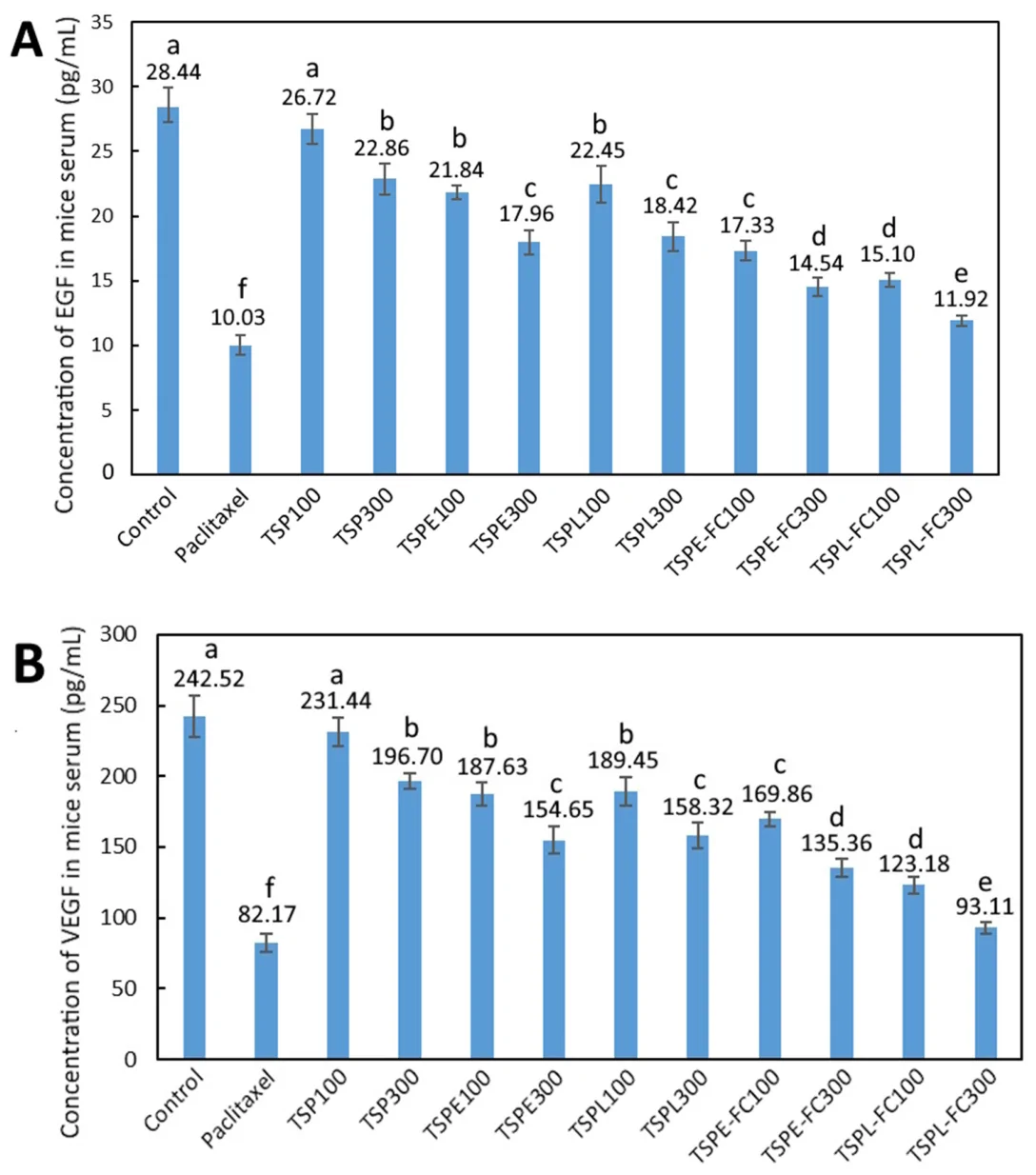
Effect of tilapia skin collagen peptides (TSP) and their nanopeptides (TSPE and TSPL) as well as folic acid-chitosan ligand conjugated nanopeptides (TSPE-FC and TSPL-FC) on serum EGF (**A**) and serum VEGF (**B**) levels in nude mice after a 3-week treatment period. TSP, TSPE, TSPL, TSPE-FC and TSPL-FC each at two different doses (100 and 300 mg/kg BW), paclitaxel at 15 mg/kg BW) and control (0.85% saline) were injected separately for a 3-week treatment period. Data are presented as mean ± standard deviation of measurements from six nude mice (*n* = 6), and the data with different small letters (a–f) are significantly different (*p* < 0.05). EGF, epidermal growth factor; VEGF, vascular endothelial growth factor; BW, body weight of nude mice.

EGF and VEGF are growth factors that play a key role in tumor development and progression through influencing cell proliferation, survival and blood vessel formation, with the former promoting tumor cell growth, invasion and metastasis, and the latter regulating angiogenesis for the formation of new blood vessels. Figure 7A,B shows the effect of different treatments on the content of EGF (A) and VEGF (B) in nude mice serum. Among all of the treatments, paclitaxel showed the highest efficiency in decreasing both EGF and VEGF levels (64.7% and 66.1%). However, for the other treatments, a slight difference was shown for EGF and VEGF. More specifically, TSPL-FC at 300 mg/kg BW was the most effective in reducing the EGF level (58.1%), followed by TSPE-FC at 300 mg/kg BW (48.9%), TSPL-FC at 100 mg/kg BW (46.9%), TSPE-FC at 100 mg/kg BW (39.1%), TSPE at 300 mg/kg BW (36.8%), TSPL at 300 mg/kg BW (35.2%), TSPE at 100 mg/kg BW (23.2%), TSPL at 100 mg/kg BW (21.1%), TSP at 300 mg/kg BW (19.6%) and TSP at 100 mg/kg BW (6.05%). On the other hand, the reduction in VEGF levels in mice serum followed the order: TSPL-FC at 300 mg/kg BW (61.6%) > TSPL-FC at 100 mg/kg BW (49.2%) > TSPE-FC at 300 mg/kg BW (44.2%) > TSPE at 300 mg/kg BW (36.2%) > TSPL at 300 mg/kg BW (34.7%) > TSPE-FC at 100 mg/kg BW (30.0%) > TSPE at 100 mg/kg BW (22.6%) > TSPL at 100 mg/kg BW (21.9%) > TSP at 300 mg/kg BW (18.9%) > TSP at 100 mg/kg BW (4.6%). Collectively, the levels of EGF and VEGF were shown to dose-dependently decrease for peptide, nanopeptide and FC ligand conjugated nanopeptide treatments, with paclitaxel, TSPL-FC/TSPE-FC and TSPE/TSPL showing the most pronounced effect, particularly at high dose (300 mg/kg BW).

### 2.7. Comparative Discussion on Antitumor Efficiency of Peptides, Nanopeptides and Folic Acid-Chitosan Conjugated Nanopeptides

Protein is a vital source of essential AA and energy for basic nutrition in the human body. Marine byproducts offer an excellent source of protein, with fish accounting for 10–25% depending on the species [21]. The fish processing industry generates an enormous amount of byproducts that are either wasted or underutilized [40]. In recent years, the isolation of value-added products such as protein hydrolysates or bioactive peptides from protein sources (collagen) has garnered great attention for possible treatment of various chronic diseases, including cancer [6,20,41]. Such bioactive peptides are oligopeptides, which are unreactive inside the protein macromolecule, but can be released by gastrointestinal digestion, fermentation and chemical/enzymatic hydrolysis [13,22]. The low-MW peptides obtained after hydrolysis are digestible and more bioavailable, and exhibit higher bioactivity than parent proteins, for possible application to treatment of chronic disease [10,11]. Compared to chemical hydrolysis, enzymatic hydrolysis offers a safe and ecofriendly approach to avoid harsh chemicals and generation of undesirable byproducts during breakdown of large proteins into low-MW bioactive peptides [13,19,22], which possess various biological functions depending on the structure, MW, AA composition and sequence.

In our earlier study, two collagen peptide fractions rich in peptide fragments at MW 482 Da and 172 Da were, respectively, produced using 2.5% and 12.5% alcalase for hydrolysis of acid-soluble collagen obtained from Taiwan tilapia skin, which were further encapsulated into nanoemulsion, nanoliposome and nanogold delivery systems for further conjugation with FC ligand for evaluation of their anticancer potential toward human lung cancer cells (A549) [24]. Of all 14 formulations, collagen peptides rich in 172 Da peptide fragments and its six nanopeptides showed a higher inhibition efficiency than collagen peptides rich in 482 Da peptide fragments and its six nanopeptides, and thus the former formulations were selected for elucidation of the inhibition mechanism. For the cell cycle study, the proportions of sub-G1, S and G2-M phases followed a dose-dependent increase, with a possible cell cycle arrest at G2/M phase, while the proportion of late apoptotic cells was the highest for nanoliposome conjugated with FC, probably through endonuclease activation for subsequent internucleosomal (deoxyribonucleic acid) DNA cleavage; necrotic cells dominated for nanoemulsion conjugated with FC through membrane damage [24]. Additionally, the ligand-conjugated nanopeptides were the most efficient in executing A549 cell apoptosis through activation of caspase-8, caspase-9 and caspase-3, followed by nanopeptide and peptide, with both external death receptor and internal mitochondria routes being involved in apoptosis of A549 cells [24]. Comparatively, nanoemulsion and nanoliposome without and with FC conjugation was shown to exert higher inhibition efficiency than nanogold, and thus in this study collagen peptides with 172 Da peptide fragments, as well as its nanoemulsion and nanoliposome before and after FC ligand conjugation, were chosen to evaluate their tumor inhibition efficiency using a nude mice model.

As mentioned before, physicochemical characteristics of bioactive peptides such as charge, hydrophobicity, AA composition and sequence, secondary structure and MW are the key determinants of their biological activity [21,40]. Low-MW peptides are reported to be more biologically active than peptides with high MW, as the cleavage of large proteins into small peptides by enzymatic hydrolysis can facilitate interaction with biological system. This enables fast absorption to elevate bioavailability while reaching target tissues more readily for exerting various biological functions [6,10]. In addition, the unique AA profile and structure of low-MW peptides enable them to bind with specific receptors or interact with specific enzymes enhancing potential biological activities [21]. Furthermore, the cell mobility and diffusivity are raised substantially with low-MW peptides. For instance, Song et al. [42] demonstrated a rise in antiproliferative activity of half-fin anchovy protein hydrolysate against several cancer cells, including prostate DU-145, lung 1299 and esophagus 109, after heating the hydrolysate to increase the proportion of low-MW peptides.

#### 2.7.1. Effect of AA Composition on Antitumor Activity

The AA composition of bioactive peptide TSP derived from Taiwan tilapia skin determined in this study was shown to contain 16.1% of essential AA, 67.2% of hydrophobic AA, 16.0% of charged AA and 2.3% of aromatic AA (Figure 1F). It is well known that essential AA are crucial building blocks for protein synthesis in the human body, playing a vital role in muscle growth, tissue repair, energy source, nutrient absorption and overall health [43]. The presence of eight essential AA (16.1%), including His (1.2%), Ile (1.3%), Leu (2.6%), Lys (2.9%), Met (1.9%), Phe (1.7%), Thr (2.3%) and Val (2.1%), in TSP from tilapia skin in this study further demonstrated the nutritional significance of this peptide product, as the human body cannot synthesize these AA (Figure 1F, Table S1).

On the other hand, it has been well documented that the presence of hydrophobic AA is responsible for various biological activities in peptides, including antioxidant, immunomodulation, anti-hypertension, antimicrobial, antidiabetes and anticancer [22,40,41,44]. In other words, the higher the proportion of hydrophobic AA in peptides, the higher the biological activity exerted by them. A total of 10 hydrophobic AA, including Ala (13.6%), Cys (0.3%), Gly (33.1%), Ile (1.3%), Leu (2.6%), Met (1.9%), Phe (1.7%), Pro (10.0%), Tyr (0.6%) and Val (2.1%), contributing to 67.2% of total AA, was present in Taiwan tilapia skin-derived TSP (Figure 1F, Table S1). Several reports have shown that endopeptidase enzymes such as alcalase, pepsin, papain and neutrase are capable of producing peptides with high proportions of hydrophobic AA for improving biological activity [6,21]. Thus, alcalase enzyme (12.5%) was selected to hydrolyze acid-soluble collagen from tilapia skin to produce TSP, which should explain the antitumor efficiency observed in nude mice in this study.

Moreover, hydrophobic AA have been reported to facilitate interaction and penetration into cancer cells, resulting in membrane disruption and cell death, as they are able to interact with the cell membrane to form pores disrupting the membrane’s integrity for subsequent release of cellular contents [21,45]. In addition, the tendency of hydrophobic AA to form helical structures can enhance cell membrane binding and interaction, thereby contributing to stability of the peptide’s structure [43]. For instance, Hsu et al. [46] produced two peptides (LPHVLTPEAGAT with 1206 Da, PTAEGGVYMVT with 1124 Da) from tuna dark muscle by using papain and protease XXIII, and demonstrated a high antiproliferative activity towards breast cancer cells MCF-7, which can be ascribed to seven hydrophobic AA, with Ala, Gly, Pro and Val being present in both peptides. More recently, Liu et al. [44] produced a low-MW protein hydrolysate (<3 KDa) containing 57.7% of hydrophobic AA from a mixture of tilapia fish head, bone and viscera, and exhibited synergistic anticancer effects in BALB/c mice by administering along with cyclophosphamide to alleviate side-effects of this drug. In another study, Zhao et al. [43] illustrated that protein hydrolysate (MW, 0.25–1 kDa) produced from tuna trimmings by alcalase hydrolysis could attenuate immune stress and intestinal mucosal injury during chemotherapy in sarcoma tumor-bearing mice, which can be attributed to a high proportion of hydrophobic AA (56%) present in the tuna protein hydrolysate. The α-helical secondary structure and aggregation of peptides were also linked to the observed antitumor effects [43]. Additionally, hydrophobic AA possess the capability to scavenge free radicals, especially lipid-derived radicals. In two separate studies, a bioactive peptide (GSTVPERTHPACPDFN) with MW 1801 Da obtained from Hoki fish frame [47], and two peptides (WAFAPA and MYPGLA) from blue spotted stingray fish [48], were shown to effectively scavenge DPPH, hydroxyl, peroxyl and superoxide radicals during lipid peroxidation, which can be attributed to the presence of hydrophobic AA in these peptides.

In addition to hydrophobic AA, the presence of charged AA in peptides also plays a vital role in bioactivity [21,41,44]. A total of five charged AA, including three positively charged Arg, His and Lys (9.5%), as well as two negatively charged Asp and Glu (11.4%) present in TSP (Figure 1F) in this study, should facilitate conjugation with the cancer cell membrane for subsequent interaction, resulting in cell apoptosis/necrosis. More elaborately, the positively charged AA can interact with the negatively charged cancer cell surface via electrostatic interactions and destabilize the membrane, causing penetration and leakage of cellular components, ultimately leading to cell death through apoptosis and/or necrosis [20,21]. Moreover, cell membrane disruption can occur via electrostatic interaction of the cancer cell surface with negatively charged phospholipid (phosphatidylserine) for subsequent rearranging of peptides in α or β-helix forms [21]. Additionally, peptides containing positively charged arginine were reported to exhibit a higher inhibitory effect towards cancer cells. For example, Song et al. [49] reported a decrease in the IC_50_ value from 11.4 mg/mL to 8.1 mg/mL after replacing Pro with Arg in the peptide YALPAH, obtained from half-fin anchovy by pepsin hydrolysis, which can be ascribed to interaction of guanidine moiety in Arg with phosphate, sulfate and carboxylate in cellular components via hydrogen bonds. Similarly, the negatively charged AA (Cys and Glu) can bind with the positive-charged regions of the cell membrane, although the net charge of the cancer cell surface is negative [40,45]. More importantly, the charged AA being either acidic or basic can exhibit antioxidant activity through their metal chelation and radical scavenging abilities [19,40]. In addition, they possess a lower tendency to induce drug resistance and selectively target cancer cells [40]. Hydrophilic AA such as Cys and His, and aromatic AA such as Phy and Tyr, were shown to scavenge free radicals, thereby decreasing the oxidative stress by donating protons [41,44]. In a recent review, Ng et al. [41] summarized a total of 16 tilapia-derived protein hydrolysates and peptides with MWs ranging from 372.42 to 6334.49 Da, concluding that a 20–100% proportion of hydrophobic AA is present, including aromatic and charged AA, and thus exerting high antioxidant activity.

#### 2.7.2. Effect of Low-MW Peptides on Antitumor Activity

Obviously, the antitumor effects observed in this study are mainly due to low-MW collagen peptides (TSP) obtained by 12.5% alcalase hydrolysis of acid-soluble collagen obtained from Tilapia skin, with a substantial improvement by passive and/or active targeting through encapsulation of TSP into nanoemulsion and nanoliposome systems without or with FC ligand. The presence of 11 peptide fragments (172–1905 Da) in TSP may have contributed either individually or synergistically to the overall antitumor effects shown (Figure 1G), with a high proportion 172 Da peptide fragments possibly exerting a dominant role in inhibiting lung tumors. Based on the MW calculation among 18 amino acids, the probable amino acid composition in the 172 Da peptide fragments was determined to be a dipeptide with either a Gly-Pro or Pro-Gly sequence, as the peptide bond formation between Gly (MW 75.07) and Pro (MW 115.13) through elimination of a water molecule (MW 18.02) resulted in a MW of 172.18 (Figure 1F,G). It is worth mentioning that Gly is the first (33.1%) and Pro is the third (10.0%) highest contributors to the total amino acid composition in TSP, with Ala (13.6%) being the second highest contributor (Figure 1F; Table S1).

It has been well documented that the biological activity of Gly-Pro/Pro-Gly dipeptides can depend on both sequence (Gly-Pro or Pro-Gly) and structure (linear or cyclic), with linear Gly-Pro/Pro-Gly being shown to possess antifibrotic and neurological effects, but without any direct evidence of their anticancer effects [50,51,52,53,54]. However, among the cyclic sequences, cyclic Gly-Pro was demonstrated to exert moderate anticancer effects, but no observable effects were reported for cyclic Pro-Gly [50,53]. For instance, cyclic Gly-Pro dipeptide was shown to inhibit liver cancer cells HepG2 and lung cancer cells A549, with an IC_50_ value at 101.8 µM and 206 µM, respectively [53]. Therefore, given the sequence and structure of the high proportion dipeptide (172 Da) in TSP, it may be postulated that cyclic Gly-Pro possessing the antitumor activity could be attributed to its encapsulation into nanoemulsion and nanoliposome systems without or with FC conjugation. Additionally, a possible synergistic effect with some other potent peptide fragments in low-MW collagen peptides (184–1905 Da) produced from Tilapia skin collagen may be responsible for this antitumor effect as well (Figure 1G).

Marine cyclic peptides have drawn great attention for over a decade owing to their low toxicity, structural diversity, high binding affinity and target selectivity, thereby possessing broad biological applications [53]. Unlike linear ones, they exhibit high membrane permeability and stability against protease enzymes. The anticancer activity of various cyclic peptides for the treatment of different types of cancers has been well documented [51]. Specifically, cyclic Gly-Pro, a prevalent marine cyclic dipeptide belonging to a group of bioactive 2,5-diketopiperazines, is a lipophilic and stable natural compound produced endogenously in the human body as a metabolite of hormone insulin-like growth factor (IGF-1) [53]. IGF-1, a 70-amino acid hormone, plays an important role in survival, growth and metabolism in all organisms, and is regulated through reversible binding to circulating and tissue-associated IGF-1 binding proteins (IGFBPs) [55]. However, both insufficient and excessive production of IGF-1 are associated, respectively, with cardiovascular/neurological disorders and tumorigenesis [50,55,56]. For the latter, IGF-1 is abundantly released by both malignant cells and the tumor microenvironment during tumorigenesis, metastasis and development of anticancer drug resistance [55]. When IGF-1 binds to its receptor, it triggers a cascade of intracellular signaling events mainly through phosphoinositide 3-kinase/protein kinase B/mechanistic target of rapamycin (PI3K/Akt/mTOR) and rat sarcoma/rapidly accelerated fibrosarcoma/mitogen-activated protein kinase/extracellular signal-regulated kinase (Ras/Raf/MAPK/ERK) pathways, thereby promoting malignant cell migration and survival by inhibiting apoptosis as well as stimulating protein synthesis, cell proliferation and differentiation by activating transcription factors [55,56]. Thus, the anticancer strategy of potential bioactive compounds can be achieved by decreasing IGF-1 levels in the tumor microenvironment.

As noted above, cyclic Gly-Pro could alter the binding of IGF-1 with its IGFBPs to regulate IGF-1 homeostasis in the tumor environment [50,53]. In a previous study, Guan et al. [50] reported that cyclic Gly-Pro could inhibit lymphomic tumor growth in mice by decreasing the excessive levels of IGF-1, while this study also demonstrated the efficacy of cyclic Gly-Pro in improving recovery from ischemic brain injury by increasing IGF-1 levels, implying that cyclic Gly-Pro could regulate the IGF-1 levels for various chronic diseases. Thus, in our study, the dominant dipeptide cyclic Gly-Pro in TSP may inhibit the A549 lung tumor growth in mice by decreasing IGF-1 levels, but this needs further investigation. Nevertheless, the observed inhibitory effects on A549 lung tumor in mice may also be related to synergistic effects of the other 11 low-MW peptide fragments (172–1905 Da) (Figure 1G), which needs further exploration in our future study. Furthermore, the possible antitumor effects of some other non-peptide bioactive compounds such as trace minerals and glycosaminoglycans co-extracted with collagen cannot be ignored.

#### 2.7.3. Effects of Nanoencapsulation on Tumor Targeting

Owing to the undesirable bitter taste resulting from the interaction of peptides rich in hydrophobic AA with taste receptors, as well as hygroscopicity, storage/textural instability and instability under gastrointestinal conditions, it is imperative to encapsulate bioactive peptides into suitable carriers or delivery system for functional food or drug development with improved bioavailability [18,26]. As nanodelivery systems including nanoemulsion, nanoliposomes and polymer nanoparticles are becoming more popular [57,58], TSP encapsulated nanoemulsion (TSPE) and nanoliposome (TSPL) were successfully prepared in this study with small particle size, high homogeneity and high stability. Following a 3-week treatment, the antitumor efficiency in terms of tumor volume improved by 1.63 to 1.80-fold for TSPE and 1.60 to 1.65-fold for TSPL (Figure 5B). Likewise, the antitumor efficiency in terms of tumor weight elevated by 1.52 to 2.34-fold for TSPE and 1.45 to 1.98-fold for TSPL (Figure 6B), implying that the encapsulation of peptides into an appropriate nanosystem can elevate their antitumor efficiency by enhanced accumulation on the tumor site through passive targeting via leaky vasculature [24,39].

Furthermore, nanoencapsulation of peptides into nanoliposomes/nanoparticles was reported to protect peptides from proteolytic enzyme degradation (proteolysis), as well as to improve various pharmacokinetic parameters through prolonging blood circulation time, reducing clearance, increasing in vivo half-life, elevating mean residence time and increasing area under the curve (AUC), thereby enhancing both tumor accumulation and therapeutic efficiency of nanoencapsulated peptides [59,60]. For instance, compared to free cyclic hexapeptide (PMX205), the half-life time, AUC and absolute bioavailability was shown to increase by 25-, 9.7- and 1.9-fold, respectively, upon oral administration of poly(lactic-*co*-glycolic)-based PMX205 nanopeptides [61]. The determination of peptide stability and pharmacokinetic parameters of free peptide (TSP) and nanopeptides (TSPE, TSPL, TSPE-FC and TSPL-FC) will be carried out in a separate study in the future.

Application of peptides possessing anticancer activity can be broadly classified into marine-derived peptides and non-marine-derived peptides. In most studies, peptides from marine and non-marine sources are explored as a stabilizer, cell targeting ligand, cell penetrating molecule along with other chemotherapeutic agents, and not by itself as an anticancer therapeutic [62,63]. Although many marine-derived peptides are reported to possess anticancer potential, their encapsulation into nanosystems to overcome the limitations of instability, low solubility, proteolytic degradation, nonspecific membrane toxicity, short circulation time and poor bioavailability still remains unexplored [64]. Some marine anticancer peptides possessing strong intrinsic anticancer activities include epinecidin-1, chrysophsin-1, pleurocidin, pardaxin and piscidin, but none of them were encapsulated into a nanodelivery system for improved therapeutic efficiency [65].

Recent advances in peptide nanomedicine have mainly focused on non-marine anticancer peptides, particularly melittin and dermaseptin, delivered through lipid nanoparticles, polymeric nanoparticles and liposomal systems [66,67]. For instance, Wang et al. [68] prepared pH-stimulus nanoliposomes with an α-helix anticancer peptide dermaseptin-pp (Der-pHSL), with particle size at 96.78 nm, polydispersity index at 0.15, zeta potential at 6.94 mV and encapsulation efficiency at 86.26% for subsequent evaluation of inhibition efficiency towards A549 cells and A549 cell-induced tumors in a nude mice model. Compared to Der-L (without pH stimulus), Der-pHSL was shown to significantly decrease haemolytic toxicity and enhance tumor cell inhibition, with high uptake in the simulated acidic tumor microenvironment. Furthermore, a significant tumor-targeting effect via EPR and pH responsiveness in the tumor environment was demonstrated in nude mice [68]. In a later study, niosomes, prepared with honeybee venom peptide melittin, with particle size at 146.2 nm, polydispersity index at 0.265 and zeta potential at 52.1 mV, were demonstrated to possess a higher anticancer efficiency towards A549 and Calu-3 cells when compared to free melittin, with IC_50_ being 0.69 and 1.02 µg/mL, respectively [69]. Additionally, upon melittin niosome treatment, Bax and caspase-3 expressions were shown to increase, respectively, by 10- and 8-fold in A549 cells and 9- and 10.5-fold in Calu-3 cells, accompanied by a 0.19- and 0.18-fold reduction in Bcl2 gene expression and inhibition of cell migration [69]. More recently, Zhang et al. [70] developed α-melittin- and resveratrol-loaded nanoliposomes with particle size, polydispersity index and zeta potential, respectively, at 66.55 nm, 0.12 and 0.45 mV, and subsequently modified with a targeting ligand angiopep-2 (Ang-α-Mel-Res-Lips) for studying their tumor inhibition effects on HS683 cell-induced glioblastoma tumor in mice. Results showed that Ang-α-Mel-Res-Lips could facilitate passage through the blood–brain barrier, increase tumor targeting, reduce α-melittin-associated hemolysis and prolong lifespan of tumor bearing mice, while α-Mel-Res-Lips could stimulate apoptosis in glioblastoma cells and induce pyroptosis-associated protein expressions, including gasdermin-D, gasdermin-E, caspase-3 and nucleotide-binding oligomerization domain-like receptor pyrin domain containing 3 (NLRP3), and inhibit epithelial–mesenchymal transition via modulating the wingless-related integration site/beta-catenin (Wnt/β-catenin) signaling pathway [70].

Several reports have also demonstrated the in vitro anticancer and/or antitumor effects of melittin following encapsulation into lipid nanocarriers. For instance, Yu et al. [71] synthesized self-assembled α-melittin-lipid nanoparticles (α-Mel-NPs) with a particle size range from 10 to 20 nm, and demonstrated their enhanced accumulation in lymph nodes with simultaneous activation of antigen-presenting cells, resulting in a 3.6-fold increase in antigen-specific CD8+ T cell responses and inhibition of primary (95%) and distant (92%) melanoma tumor growth in mice induced by B16F20 cells. Likewise, in a later study, melittin-loaded and hyaluronic acid-conjugated phospholipid scaffold nanoparticles (Mel-HAPS-NPs) were synthesized by an electrostatic interaction method, with particle size at 45.1 nm and zeta potential at 6.27 mV [72]. Additionally, Mel-HAPS-NPs were shown to exert dual-targeting effects on both 4T1 cell-induced breast tumor in mice and its metastatic sentinel lymph nodes, as evident by an inhibition efficiency of 81.3% and 78.0%, respectively, compared to the control (phosphate buffered saline (PBS)-treated mice). To overcome the nonspecific hemolytic activity, intrinsic instability and immunogenicity, Lv et al. [73] developed melittin micelles with poly(ethylene glycol) methyl ether (4-cyano-4-pentanoate dodecyl trithiocarbonate), 2-diisopropylaminoethyl methacrylate and pyridyl disulfide ethyl methacrylate by using D-melittin instead of the frequently used L-melittin. The synthesized D-melittin micelles, with particle size at 32.6 nm and critical micelle concentration at 0.03 mg/mL, were shown to exhibit significant cytotoxicity against breast cancer cells 4T1 and colon cancer cells CT26, as well as antitumor effects in 4T1-induced and CT26-induced tumors in mice. In some recent studies, polymeric melittin nanoparticles have also been reported to be capable of releasing melittin in a cooling-induced tumor microenvironment in colon26 NL-17 bearing mice [74], as well as effective in exerting synergistic antitumor effects with photodynamic therapy on breast cancer cells 4T1-induced tumor-bearing mice [75]. Two recent review articles have summarized the therapeutic efficiency and mechanisms associated with melittin-based nanocarriers in cancer therapy [66,67].

Collectively, the foregoing discussion revealed that the number and variety of peptides encapsulated into nanosystems for evaluation of antitumor activity is limited. To the best of our knowledge, only several non-marine peptides have been encapsulated into nanoliposome and lipid/polymeric nanoparticles, while no studies report antitumor efficiency of peptides encapsulated into nanoemulsion and folic acid-conjugated peptide nanosystems.

#### 2.7.4. Effects of Ligand Conjugation on Tumor Targeting

Both TSPE and TSPL were further conjugated to folic acid-chitosan ligand to obtain TSPE-FC and TSPL-FC, both of which were shown to exert a much higher antitumor effect. Compared to TSPE and TSPL, the antitumor efficiency in terms of tumor volume rose by 1.11- to 1.46-fold after conjugating FC with TSPE (TSPE-FC), and by 1.24- to 1.94-fold after conjugating FC with TSPL (TSPL-FC) (Figure 5B). For tumor weight, it was increased by 1.09- to 1.39-fold for TSPE-FC and 1.22- to 1.97-fold for TSPL-FC (Figure 6B). This finding verified that FC conjugation with TSPE and TSPL enhanced their accumulation on the tumor site by active targeting for improved antitumor activity.

Folic acid, a water-soluble vitamin B9, consisting of three major components including glutamic acid, para-aminobenzoic acid and heterocyclic pteridine ring [7], is a vital component for normal cell metabolism, DNA synthesis and repair. However, as cancer cells rapidly divide, they require a high level of folic acid to maintain DNA synthesis. Consequently, compared to normal cells, folate receptors, especially the α-form, are overexpressed 100–300 times higher in most cancer cells [8,29]. When folic acid-conjugated nanoparticles bind to folate receptors on the tumor cell surface, they rapidly penetrate into the cells through receptor-mediated endocytosis and are internalized to form intracellular endosomes. After internalization of the nanoparticles, folate receptors detach from the acidic tumor environment and return to the cell surface, while folic acid-conjugated nanoparticles undergo degeneration by lysosome for subsequent release into the cytosol, which allows the localization of nanoparticles through their selective delivery to the tumor site, thereby minimizing side-effects [7,8,76]. Thus, folic acid, being nontoxic, low MW, easy to synthesize, stable and nonimmunogenic, is the most widely used tumor-targeting ligand compared to antibodies [29].

On the other hand, chitosan, a linear polysaccharide formed by chitin deacetylation in alkaline medium, is an abundantly available natural biopolymer with widespread applications in tissue engineering and drug delivery [77]. Both amino and hydroxy functional groups present in chitosan make it a promising candidate for conjugation to cancer-targeting ligands, including folic acid and peptides. In addition, it helps in transporting the bioactives to the acidic tumor environment. Moreover, chitosan alone was shown to exert antitumor activity through electrostatic interaction with the negatively charged cancer cell surface, resulting in membrane disruption and apoptosis [27]. Thus, owing to its biodegradable, biocompatible, nontoxic, low immunogenic and cationic nature [8], chitosan is an excellent biomaterial for coating and conjugation purposes. For conjugation of folic acid with chitosan, EDC reagent was used to facilitate activation of the carboxylic acid group in folic acid to be more reactive for the formation of an amide bond by the carbodiimide coupling reaction with the amine group in chitosan [29,78].

Some recent review articles have emphasized the growing importance of folic acid conjugated nanodelivery systems for application in cancer therapy [7,8,28,58]. Additionally, folic acid-chitosan conjugates were shown to enhance the anticancer effect of therapeutic drugs such as sorafenib [77] and cytarabine [29], as well as act as nanocarriers of natural bioactive compounds such as α-mangostin [27], resveratrol, curcumin and genistein [79]. Taken together, the foregoing discussion corroborates the highest antitumor efficiency of TSPE-FC and TSPL-FC observed in a nude mice model in this study. Thus, it may be hypothesized that FC conjugated to TSPE and TSPL should enable high tumor targeting by enhanced accumulation through effective interaction with folate receptors highly expressed in tumor cells. However, the FC-mediated targeting needs to be confirmed by determination of FOLR1 expression and receptor-dependent uptake or biodistribution in a future study.

Although both TSPE-FC and TSPL-FC developed in this study can achieve tumor-targeting effect, the challenge still exists regarding the amount of nanoparticles accumulated in the tumors, which needs further exploration. However, it has been demonstrated that direct affinity targeting of malignant cells with homing peptides, a short sequence of amino acids that specifically bind to unique receptors on the surface of cancer cells, has been proven to be a successful strategy for treating tumors [63,80,81]. Among the various nanosystems, liposomes were shown to reduce systemic toxicity, increase biodistribution, prolong blood circulation and thereby elevate tumor accumulation [82,83]. However, these liposomes failed to improve overall survival rate when compared with the doxorubicin therapy, which can be attributed to the absence of a targeting ligand conjugated with liposome. Thus, in our study, both TSPE-FC and TSPL-FC should be more efficient in achieving a tumor-targeting effect than nanoemulsions/nanoliposomes without conjugation with targeting ligands. Furthermore, conjugating homing peptides to nanoparticles was shown to enhance their binding strength to target cancer cells and thereby improve both specificity and therapeutic efficiency [80]. Based on a recent review report by Sidorenko et al. [80], tumor-targeting peptides can be further classified into tumor-penetrating peptides and vascular docking (homing) peptides, with the former being capable of promoting extravasation and deep penetration into the tumor parenchyma following the initial recruitment to activate biological pathways. The latter remained confined to the vascular endothelial barrier for the specific interaction between the peptide and its vascular receptor, and limited the penetration of high-affinity molecular like antibodies into solid tumors. This classification should provide a clearer elucidation of the possible peptide mechanisms in the treatment of tumors. Additionally, the advantages of short peptides should eliminate the need for complicated antibody engineering, provide cost efficiency, enable straightforward synthesis and interact with functionally important binding pockets on the surface of target molecules [80].

#### 2.7.5. Tumor Cell Internalization of Peptides and Nanopeptides

In this study, the mean particle sizes of TSPE, TSPL, TSPE-FC and TSPL-FC were 16.1, 33.9, 15.2 and 35.3 nm, respectively, which should be able to penetrate into the cytoplasm and nucleus from the extracellular matrix, achieving antitumor effects, as the mean size of human cells is 10–100 µm [84]. Furthermore, for TSPE and TSPL without conjugation with ligand, the nanoparticle accumulation in the tumor tissue is a passive process requiring long circulation to speed up the time-dependent extravasations through the EPR effect. The endothelial cells in the blood vessels of normal tissues were reported to have gaps which are about 7–10 nm in size, but may increase to a few hundred nanometers in the tumor microvasculature for subsequent nanoparticle accumulation [39]. Most importantly, the eradication of nanoparticles in TSPE, TSPL, TSPE-FC and TSPL-FC from the reticuloendothelial system was avoided with nanoparticle sizes < 200 nm [39]. As the low-MW collagen peptides (172-1905 Da) prepared in our study are composed of 18 amino acids with a high proportion of 172 Da peptide fragments, they should be able to penetrate into the cell membrane more effectively for non-targeted and targeted therapy. Collectively, from the foregoing discussion, it may be deduced that injected nanopeptides could be accumulated and internalized into tumor cells, as FC conjugated and nanoencapsulated peptides are capable of accumulating in the tumor environment via both passive and active targeting with effective internalization into tumor cells.

However, in this study, we only measured tumor volume and weight, as well as VEGF and EGF in mice serum, which should be inadequate. Instead, in our future study, it is imperative to perform qualitative and quantitative analyses of nanopeptides in tumor distribution for confirmation of nanopeptide accumulation in tumor cells and histopathological examination to detect cellular- and tissue-level changes for elucidation of both therapeutic efficiency and treatment safety.

#### 2.7.6. Effect of Peptide/Nanopeptide Treatments on EGF/VEGF Levels in Mice Serum

EGF and VEGF are two critical biomarkers involved in tumor progression, and are frequently used to evaluate the therapeutic efficacy of anticancer peptides including peptide-based therapeutics [85]. EGF stimulates tumor cell proliferation through activation of the epidermal growth factor receptor (EGFR), leading to downstream activation of signaling pathways that promote cell survival, proliferation, migration and resistance to apoptosis [86]. Similarly, VEGF acts as the principal regulator of tumor angiogenesis by stimulating endothelial cell proliferation and neovascularization through vascular epidermal growth factor receptor-2 (VEGFR-2) signaling, thereby supplying tumors with oxygen and nutrients necessary for continuous growth and metastatic propagation [87]. Overexpression of both EGF/EGFR and VEGF has been strongly correlated with rapid tumor growth, angiogenesis, invasion, metastasis and poor prognosis in numerous cancers [85]. Consequently, suppression of these biomarkers is widely regarded as evidence of effective antitumor therapy.

In addition to evaluating the antitumor efficiency of tilapia skin peptides and nanopeptides in this study, both EGF and VEGF levels were also determined in mice serum by using commercially available kits. Compared to control, all of the treatments were shown to dose-dependently decrease the levels of EGF and VEGF in mice serum, with TSPL-FC showing the most pronounced effect, followed by TSPE-FC, nanoemulsion and nanoliposome without conjugation, and peptide (Figure 7). The dose-dependent decrease in EGF levels may indicate reduced tumor size following an increase in treatment dose. Similarly, the observed dose-dependent decrease in VEGF indicates a possible inhibition of tumor angiogenesis with a concomitant reduction in tumor size with increasing treatment dose. VEGF-mediated angiogenesis is essential for tumors to acquire an adequate blood supply that supports sustained growth and metastatic propagation [87]. A reduction in VEGF expression suppresses endothelial cell activation and neovascularization, thereby limiting oxygen and nutrient delivery to tumor tissues, and creating an unfavorable microenvironment for tumor progression [85]. Such decrease in growth factors has been recognized as one of the principal molecular mechanisms underlying the anticancer activity of many marine-derived peptides [9,65,88]. Unlike conventional chemotherapeutic agents that frequently target a single molecular pathway, the concurrent dose-dependent reduction in both EGF and VEGF observed in this study suggests dual-targeted antitumor activity involving inhibition of both tumor proliferation and angiogenesis, which is the hallmark characteristic of peptide-based anticancer therapeutics [9,65,86]. Nevertheless, the mechanism of suppression of the tumor signaling pathway needs to be confirmed in a future study.

#### 2.7.7. Biocompatibility, Immunogenicity, Biodegradability, Allergenicity and Antigenicity of Collagen Peptides

It is well known that collagen is one of the major components of connective tissue, with collagen type I being commonly found in the body as a part of cartilage, bone, dentin and cell architecture. More importantly, collagen has been shown to be biocompatible and possess high water absorption capacity with low immunogenicity and biodegradability, and thus fish collagen has gained enormous popularity in the health and cosmetic industry due to a lower risk of unwanted immunogenicity and transmission of zoonotic infections [89]. Thus, it is pivotal to determine the biocompatibility of fish skin-based collagen and peptides, aiming for a selective cytotoxicity towards cancer cells while retaining a high viability for normal cells. In our previous in vitro study, all nanopeptides showed a higher cytotoxicity towards A549 cells than normal lung cells MRC5, while a high cell viability was maintained for the latter [24], revealing the potential application of nanopeptides in the treatment of lung cancer with minimal side effects. Zhang et al. [90] also showed the in vitro biocompatibility of type I collagen from tilapia fish skin by demonstrating high cell proliferation towards human foreskin fibroblast (HFF-1) cells, with both pepsin-soluble collagen (PSC) and acid-soluble collagen (ASC) showing high blood compatibility by hemolysis test. In another study, Zhang et al. [91] demonstrated no toxicological effect in terms of respiratory illness, abdominal irritation, eye lid and prolapse, behavioral activity and survival rate in mice treated with tilapia fish skin-derived type I collagen.

Furthermore, both biodegradability and immunogenicity are the critical issues that need to be addressed for application of fish skin-derived collagen and peptides in the biomedical field. In a study dealing with comparative biodegradability of bovine tendon collagen and tilapia collagen implanted into male rabbits, Zhang et al. [90] reported a time-dependent decline in the size of implanted collagens with a subsequent disappearance after 8 weeks, accompanied by a complete granulation of tissue in the implant position. In the same study, compared to bovine tendon collagen, type 1 collagen from tilapia skin was demonstrated to produce a much lower level of cytokines (IL-4, IL-6 and TGF-β), implying low immunogenicity of the latter [90]. However, in a clinical trial dealing with investigation of the relevance of fish collagen as an allergen in a large patient population (*n* = 101), 21% of 101 patients were sensitized to collagen, with 8 collagen-sensitized patients demonstrating absence of parvalbumin-specific immunoglobulin E (IgE) to some fish species [92]. In addition, most of the collagen-sensitized patients’ sera contained IgE binding to parvalbumins, a major fish allergen, which is highly cross-reactive [92]. However, the fish processing method may lead to an increase, decrease or change in fish allergenicity and antigenicity caused by the change of protein denaturation, protein solubility and the modification of linear or conformational epitopes [93]. For instance, the immunoglobulin G (IgG) immunoreactivity decreased when fish was processed into surimi, while trypsin hydrolysis generated two polypeptide fragments from cod that are allergenic [94,95]. An important finding is that the employment of non-thermal processing techniques such as high hydrostatic pressure could alter the secondary and tertiary structure of parvalbumin, which could reduce its antigenicity effectively [96]. As a mild enzymatic hydrolysis condition (50 °C, 6 h) was used in our study to obtain tilapia skin with low MW, the allergenicity could be reduced substantially.

Inflammation, a normal immune response of the body’s innate and adaptive immune systems to infections, can protect the body from injury due to external toxins and stimuli. However, the attack of inflammatory factors can lead to cellular apoptosis or necrosis and reduction of immune functions, resulting in tissue/organ dysfunction [97]. In a recent review dealing with the application and mechanism of bioactive peptides in the treatment of inflammation, Liu et al. [97] concluded that bioactive peptides could provide anti-inflammation effects with minimal side effects through immunomodulatory mechanisms such as reduction of oxidative stress, regulation of the release of inflammatory mediators and modulation of MAPK and nuclear factor kappa light chain enhancer of activated B (NF-κB) signaling pathways.

#### 2.7.8. Human-Equivalent Dose and Maximum Recommended Starting Dose for Clinical Study

It has been well documented that choosing a safe and effective dose of a drug is of great concern for animal experiments and human trials [98]. Additionally, an accurate dose conversion from animal to human is pivotal for estimation of human-equivalent dose (HED) and subsequent determination of maximum recommended starting dose (MRSD) for the first clinical study [99]. From the clinical safety point of view, the common practice of scaling the dose based on the body weight alone is not a correct approach. Instead, the extrapolation of dose from animal to human requires information on the pharmacokinetics, metabolic functions, body surface area and physiological time [98]. Among several methods, the dose conversion by a factor method is an empirical approach that requires no observed adverse effect levels (NOAEL) of a drug from preclinical toxicological studies (dose used in animal experiments) to estimate body surface area based HED (HED_SA_) according to the formula shown below [98,99]:

(1) $${HED}_{SA}(mg/g)=Animal\;dose\;(mg/g)\times {\left(\frac{{Weight}_{animal}(kg)}{{Weight}_{human}(kg)}\right)}^{1-0.67}$$

where 0.67 is the exponent for body surface area that accounts for the difference in metabolic rate between animal and human. Based on Equation (1), the HED for two animal doses (100 and 300 mg/kg) used in this study was calculated to be 7.12 mg/kg and 21.36 mg/kg, respectively. Thus, for a 60 kg human, a dose of 427.2 mg and 1281.6 mg is required, respectively. Likewise, for the animal dose of 15 mg/kg for paclitaxel, the HED was calculated to be 1.07 mg/kg with the human dose being 64.2 mg.

Furthermore, body weight is an equally important factor to be considered for dose conversion between animal and human. Based on the correction factor (K_m_) obtained by dividing mice body weight (0.02 kg) by surface area (0.007 m^2^) and human body weight (60 kg) by surface area (1.62 m^2^), the HED based on body weight (HED_W_) can be calculated using the formula shown below [98,99]:

(2) $${HED}_{W}(mg/kg)=Animal\;dose\;(mg/g)\times \left(\frac{Animal\;K_{m}}{Human\;K_{m}}\right)$$

By using Equation (2), the HED_W_ was calculated to be 7.71 mg/kg and 23.14 mg/kg, respectively, for 100 mg/kg and 300 mg/kg animal dose used in this study. Thus, a dose of 462.60 mg and 1388.4 mg is required for human (60 kg), respectively. Likewise, for paclitaxel with animal dose of 15 mg/kg used in this study, the HED_W_ was calculated to be 1.16 mg/kg, which is equivalent to 69.6 mg for human. Comparatively, as the HED_SA_ (427.2 mg and 1281.6 mg) was relatively lower than the HED_W_ (462.60 mg and 1388.4 mg), the former was chosen to estimate the MRSD by dividing with a factor of 10 for safety concerns [98,99]. Thus, for low and high doses (100 and 300 mg/kg) used in this animal study, the MRSD value was calculated to be 42.72 mg and 128.16 mg, respectively, with the latter being the entry-level dose (1281.6 mg) for the first clinical trial in human. The calculated high HED_SA_ dose in this study (1281.6 mg) is practically attainable through diet or dietary supplement intake, as the effective amounts of functional collagen peptide (2.5 to 15 g per day) reported in the literature have been demonstrated to be below the maximum collagen level (16 to 20 g per day) that may be incorporated in the standard American diet [100].

## 3. Materials and Methods

### 3.1. Production of TSC and TSP from Tilapia Skin and Their Characterization

Both TSC and TSP were prepared based on the methods reported in our previous study [24]. Initially, TSC was prepared by pretreatment of freeze-dried tilapia skin with 0.5 M NaOH and 0.5 M CH_3_COOH, followed by protein precipitation using 2.6 M NaCl and purification by dialysis in CH_3_COOH and deionized water. Then, TSC was subjected to enzymatic hydrolysis using an optimized condition of alcalase (Sigma-Aldrich, St. Louis, MO, USA) at 12.5%, sample-to-water ratio at 1:30 (*w*/*v*), pH at 8, temperature at 50 °C and hydrolysis time at 6 h as reported in our previous study [24], and the degree of hydrolysis was determined [16]. Both TSC and TSP were subsequently characterized for collagen type and MW by SDS-PAGE [17,24], surface charge by Horiba zeta potential analyzer (Kyoto, Japan) and particle size and morphology by SEM (TM4000 Plus model, Hitachi High-Tech Taiwan Corp., Taipei, Taiwan) [24]. Detailed procedures for peptide production and characterization of TSC and TSP by SDS-PAGE, zeta potential and SEM analyses are provided in the Supplementary Materials.

Furthermore, spectroscopic analyses for peptide characterization were carried out by measurement of absorption spectra by UV-visible spectrophotometer (U1900 model Hitachi, Tokyo, Japan), functional groups by FTIR (FT730 model, Horiba, Kyoto, Japan), amino acid composition by HPLC-DAD (1200 series, Agilent, Palo Alto, CA, USA) and MW distribution by MALDI-TOF-MS (Bruker, Billerica, MA, USA). To obtain the absorption spectra, 10 mg TSC and TSP sample solutions in 0.1 M CH_3_COOH were separately prepared and analyzed in the ultraviolet wavelength range of 200–400 nm (2 nm/min) [16,17]. FTIR spectra was recorded by separately grinding TSC and TSP samples with KBr crystals (1:100, *w*/*w*) using a mortar/pestle, pelletizing at 8000 psi and measuring in the frequency range of 4000–400 cm^−1^ [24]. The amino composition of TSC and TSP was determined based on a method issued by Taiwan Food and Drug Administration (TFDA) [101]. Initially, the sample was hydrolyzed by mixing 0.2 g of TSC or TSP with 6 mL of 6 N hydrochloric acid in vacuum-sealed glass tubes at 110 °C for 24 h, vaporizing under reduced pressure at room temperature and dissolving in 2 mL of 0.4 M boric acid. For derivatization of amino acids, the hydrolyzed TSC, TSP or amino acid standard solution was mixed with 100 μL of 0.4 M boric acid and 20 μL of the ortho-phthalaldehyde (OPA) reagent prepared by dissolving 100 mg of OPA in 9 mL of 0.4 M boric acid, followed by adding 20 μL of 3-mercaptopropionic acid and diluting to 10 mL with 0.4 M boric acid. After vortexing the mixture for 1 min, 20 μL of freshly prepared 9-fluorenylmethyloxycarbonyl (FMOC) solution (20 mg of FMOC in 10 mL acetonitrile) was added, vortexed for 30 s, diluted with 1.28 mL of deionized water, filtered through a 0.2 μm polyvinylidene fluoride (PVDF) membrane filter and 10 μL injected into HPLC-DAD for analysis. By employing a Poroshell HPH-C18 column (100 × 3 mm ID, particle size 2.7 μm) and a gradient mobile phase of 0.55% monosodium phosphate in water (pH 7.8) (A) and acetonitrile/methanol/water (45:45:10, *v*/*v*/*v*) (B), 18 amino acids were separated within 16.2 min with flow rate at 0.5 mL/min, column temperature at 40 °C and detection wavelength at 262 nm and 338 nm. The gradient condition was 93% A and 7% B initially, maintained for 0.5 min, changed to 75% A and 25% B in 5 min, 72% A and 28% B in 7 min, 54% A and 46% B in 12 min, 100% B from 13 min to 16 min, 93% A and 7% B in 16.1 min and maintained until 20 min. Identification of individual amino acids in hydrolyzed samples was based on comparison of retention time of peaks in hydrolyzed samples with that in amino acid standards. Quantitation of 18 amino acids including alanine, arginine, aspartic acid, cystine, glutamic acid, glycine, histidine, isoleucine, lysine, leucine, methionine, phenylalanine, proline, serine, threonine, tyrosine, valine and hydroxyproline (Sigma-Aldrich, St. Louis, MO, USA) was done by preparing the calibration curves with 7 different concentrations of each amino acid (0.083, 0.125, 0.250, 0.417, 0.625, 1.250 and 2.500 μM), filtering through a 0.2 µm PVDF membrane filter and injecting 10 µL into HPLC-DAD for analysis. The content of each individual amino acid was then calculated using the formula shown below [101]:

(3) $$\mathrm{Amino}\;\mathrm{acid}\;\mathrm{content}\;(\mathrm{mg}/100\;g)\;=\;\frac{C\times V}{M\times 10}$$

where C is the concentration of the individual amino acid (μg/mL) based on the standard curve, V is the final constant volume of the sample (25 mL) and M is the sample weight in grams.

For MW distribution analysis by MALDI-TOF-MS, a 1 mg TSP sample was dissolved in 1 mL of 0.1% CF_3_COOH in deionized water and desalted by zip-tip method through pipetting and discarding 10 times sequentially with 10 µL of 0.1% CF_3_COOH in acetonitrile-deionized water (1:1, *v*/*v*) (wetting solution) and 10 µL of 0.1% CF_3_COOH in deionized water (equilibration solution). After absorbing/dispensing the sample solution 10 times, the zip-tip was washed using equilibration solution, followed by consecutively eluting the sample with 3 different proportions of acetonitrile in deionized water (30, 50 and 70%) 10 times separately for collection of various eluates in Eppendorf tubes. Then, 1 µL of each collected eluate and 1 µL of α-cyano-4-hydroxycinnamic acid were then loaded on the sample plate of MALDI-TOF-MS for analysis with positive ion mode in the mass-to-charge (*m*/*z*) detection range from 0 to 1500, laser wavelength at 355 nm and laser frequency at 0.001–10 kHz [24].

### 3.2. Preparation of Tilapia Skin Collagen Peptide Nanoemulsion (TSPE) and Nanoliposome (TSPL)

Based on a method reported by Inbaraj et al. [24], the TSPE was freshly prepared by dissolving 1.0 g sample of TSP in deionized water (0.5 g) and sequentially adding soybean oil (1.0 g), lecithin (0.5 g), glycerol (0.2 g), Tween 80 (5.0 g) and deionized water (42.8 g) with a 3 min mixing after each component addition. This mixture was then sonicated in a tank ultrasonicator for 30 min and further sonicated using a probe-type ultrasonicator in an ice bath for 2 min with a 10 s pause between each 15 s sonication to obtain 50 mL of TSPE containing 20,000 µg/mL of TSP.

Following a method reported by Inbaraj et al. [24], the TSPL was freshly prepared by dissolving 0.1 g of cholesterol in 99% ethanol (25 mL) and consecutively adding phosphatidylcholine (1.0 g), Tween 80 (1.0 g) and glycerol (0.2 g) in a round-bottom flask, followed by mixing thoroughly and evaporating under vacuum (55 °C) to obtain a thin-film. Meanwhile, deionized water (47.7 g) containing TSP (1.0 g) was preheated at 60 °C in a falcon tube and added to dissolve the thin-film. After vortexing for 5 min, multilamellar vesicles were formed, which were then subjected to probe-sonication in an ice-bath for 2 min with a 10 s pause between each 15 s sonication to acquire 50 mL of TSPL containing 20,000 µg/mL of TSP.

### 3.3. Preparation of Folic Acid-Chitosan (FC) Ligand Conjugated TSPE and TSPL (TSPE-FC and TSPL-FC)

Both TSPE-FC and TSPL-FC were prepared separately based on a method reported by Inbaraj et al. [24] by mixing folic acid (0.25 g) and N-(3-dimethylamino propyl)-N′-ethylcarbodiimide hydrochloride (0.1 g) in 0.1 M NaOH (25 mL) and stirring for 3 h. Meanwhile, 25 mL of 1% chitosan in CH_3_COOH was prepared, added to the above mixture and stirred for 12 h to obtain FC ligand. Then, 50 mL of FC ligand was mixed separately with 50 mL of 20,000 µg/mL TSPE and 20,000 µg/mL TSPL each, followed by a 12 h stirring to obtain 100 mL of TSPE-FC and 100 mL of TSPL-FC, with each containing TSP at 10,000 µg/mL and folic acid at 1.25 mg/mL.

### 3.4. Characterization of TSPE, TSPL, TSPE-FC and TSPL-FC

Both particle size and PDI were measured in a Brookhaven DLS analyzer (Holtsville, NY, USA) by separately diluting TSPE and TSPL 100-fold with 25 mM KH_2_PO_4_ (pH 5.5) in a 2 mL disposable cuvette. However, a 200-fold dilution with 25 mM KH_2_PO_4_ (pH 5.5) was required for TSPE-FC and TSPL-FC to prevent the interference by FC ligand and obtain a more diluted solution for measurement by DLS. For visualizing the morphology, TEM images were obtained by diluting each sample 50-fold with deionized water and dropping on a copper grid. After 90 s, the surplus sample was removed using a filter paper, followed by adding phosphotungstic acid (20 µL) for negative staining (90 s), removing surplus stain using a filter paper and drying in a desiccator for 24 h for imaging using a JEM-1400 JEOL TEM instrument (Tokyo, Japan) at 120 kV. The zeta potential was measured by diluting 20 µL of each sample 100-fold with deionized water and pouring 300 µL into a folded capillary cell.

The encapsulation efficiency of TSP in TSPE was determined based on a method reported by Inbaraj et al. [24] with some modifications. A 250 µL TSPE sample was initially diluted with 500 µL of deionized water, followed by pouring into a centrifuge tube fitted with 3 kDa dialysis membrane, centrifuging at 12,000 rpm for 30 min at 4 °C and collecting 250 µL of free peptides from the top layer. After evaporation to dryness under nitrogen, the residue was redissolved in deionized water and 50 µL of sample mixed with 100 µL of bicinchoninic acid/copper reagent solution for subsequent incubation at 37 °C for 30 min and measuring absorbance at 562 nm. By adopting the same method, a calibration curve was prepared with different concentrations of bovine serum albumin ranging from 100 to 6400 µg/mL. The percentage of encapsulation efficiency was then calculated based on the amount of peptide originally used for TSPE preparation.

For determination of encapsulation efficiency of TSP in TSPL, a method based on Kung et al. [25] was used with a slight modification. TSPL sample (250 µL) was mixed with 0.5 mL of acetone in a centrifuge tube and centrifuged at 12,000 rpm for 30 min at 4 °C to facilitate separation of free peptides (top layer) from peptide-encapsulated nanoliposomes (bottom layer). Then, the supernatant was collected and evaporated in an oven at 60 °C for complete removal of solvent, followed by redissolving the residue in deionized water (250 µL) and determining the concentration of free peptides in TSPL. In addition, the total peptide content in TSPL was measured by mixing a 250 µL TSPL sample with 0.5 mL of 0.06% Triton for breakdown of lipid bilayer and subsequent determination of total TSP in TSPL using the bicinchoninic acid protein assay as described above.

### 3.5. In Vitro Release Study

The in vitro release study was conducted to elucidate the stability and release kinetics of TSP from TSPE, TSPL, TSPE-FC and TSPL-FC by using the dynamic dialysis method reported by Sharma et al. [34] with a slight modification. Briefly, 2 mL of each sample was poured separately into a dialysis bag (12 kDa MW cutoff) and immersed in a 50 mL PBS release medium (pH 7.4) at 37 °C with stirring at 100 rpm. After predetermined time intervals (0, 0.5, 1, 2, 4, 6, 8, 12, 18, 24, 36 and 48 h), 1 mL of PBS was collected for analysis of released peptide by bicinchoninic acid protein assay as described above, and 1 mL of fresh PBS was replaced to maintain a constant volume. The cumulative release in percentage was determined based on the released TSP content at each specific time interval (C_T_) and the initial free TSP/encapsulated TSP (C_I_) by using the formula,

(4) $$\mathrm{Cumulative}\;\mathrm{release}\;(\%)=\frac{C_{T}}{C_{I}}\times 100$$

### 3.6. Animal Experiments

Initially, an approval for conducting designed experimental protocols was obtained from the Institutional Animal Care and Use Committee (IACUC Approval number A11237, dated 9 April 2024). An approved guideline for ethical conduct in the care and use of animals recommended by the American Physiological Association (APA) was followed [102]. Seventy-two 4-week-old male nude mice, each weighing about 20 g, were procured from BioLASCO (Taipei, Taiwan) and housed in Fu Jen Catholic University Animal Research Center in ventilated cages and provided with sterilized water and standard laboratory rodent chow ad libitum (Lab Diet 5001). Moreover, a 12 h light/dark cycle was maintained in the animal room with a temperature of 22 ± 2 °C and relative humidity of 55 ± 10% controlled throughout the experiments. In addition, environmental conditions including lighting, noise levels, air quality, humidity and temperature were standardized across all cages throughout the experiment. To reduce stress and promote natural behavior, environmental enrichment in the form of wooden sticks, bedding materials and sterilized water were provided and replenished every week. The body weight of all of the mice was measured twice a week. One mouse was considered as one experimental unit and the mice were randomly assigned to individual standard polycarbonate cages with stainless steel wire lids (two mice per cage) by the animal center personnel for subsequent numbering with one blank control group first, followed by one positive control paclitaxel group and ten peptide/nanopeptide treatment groups. To minimize potential confounders on experimental outcome, both numbering order and cage position were maintained in an open rack, and all researchers involved in this study were informed of group allocation and order of cages during different stages of experiment including allocation, conduction of experiment, outcome assessment and data analyses. An animal center personnel was dedicated to monitoring the overall health of mice once in two days. To reduce handling stress, mice were gently grasped by the tail and supported with the opposite hand while removing from the cage and subsequently calmed by gentle stroking before feeding. Additionally, by using a subcutaneous injection of ketoprofen (2–3 mg/kg), the obvious signs of pain in mice including social withdrawal/self-isolation, vocalization and aggressive/defensive behavior were effectively managed. Prior to start of experiments, it was established to euthanize mice by CO_2_ inhalation if the humane endpoints including severe weakness with an inability to eat/drink, visible damage to vital organs and rapid weight loss were encountered. Nevertheless, such adverse events did not occur during the animal experiments in this study.

#### 3.6.1. Determination of Antitumor Efficiency

Initially, human lung cancer cells A549 (ATCC-CCL-185) procured from the Bioresource Collection and Research Center (BCRC), Taiwan Food Industry Research and Development Institute (Hsinchu, Taiwan) were cultured in 90 % Ham’s F12K medium with 2 mM L-glutamine adjusted to contain 1.5 g/L sodium bicarbonate and 10 % FBS. After an initial adaptation period of one week for mice, about 5 × 10^6^ human lung cancer cells A549 were injected into the mice flank for tumor induction. By using a vernier caliper, the tumor size was measured twice a week and the tumor volume was calculated by substituting length (L, mm) and width (W, mm) of tumor in the formula, (L × W^2^)/2 [38,39]. When the tumor volume attained ~250 mm^3^ 3 weeks after induction, all 72 mice were divided into 12 groups (*n* = 6) and administered with the following treatments via intraperitoneal injection for 3 weeks: Group 1 (control) injected with 0.85% saline, Group 2 (positive control) injected with 15 mg/kg body weight (BW) paclitaxel drug, Group 3 (TSP100) injected with a low TSP dose of 100 mg/kg BW, Group 4 (TSP300) injected with a high TSP dose of 300 mg/kg BW, Group 5 (TSPE100) injected with a low TSPE dose of 100 mg/kg BW, Group 6 (TSPE300) injected with a high TSPE dose of 300 mg/kg BW, Group 7 (TSPL100) injected with a low TSPL dose of 100 mg/kg BW, Group 8 (TSPL300) injected with a high TSPL dose of 300 mg/kg BW, Group 9 (TSPE-FC100) injected with a low TSPE-FC dose of 100 mg/kg BW, Group 10 (TSPE-FC300) injected with a high TSPE-FC dose of 300 mg/kg BW, Group 11 (TSPL-FC100) injected with a low TSPL-FC dose of 100 mg/kg BW and Group 12 (TSPL-FC300) injected with a high TSPL-FC dose of 300 mg/kg BW. As no mouse died during the study, all data analyses were conducted with 6 mice per group.

Positive control drug paclitaxel treatment at 15 mg/kg BW dose in Group 2 was conducted by injecting 0.3 mL of 1000 µg/mL paclitaxel solution per mouse. For TSP, TSPE and TSPL treatments, an injection volume of 0.1 mL and 0.3 mL of each formulation at 20,000 µg/mL was injected per mouse, respectively, for low dose treatments (Groups 3, 5, 7) and high dose treatments (Groups 4, 6, 8). However, an injection volume of 0.2 mL of 10,000 µg/mL was injected per mouse once every two days for low dose of TSPE-FC treatment in Group 9 and TSPL-FC treatment in Group 11, while for high dose of TSPE-FC (Group 10) and TSPL-FC (Group 12) treatments, 0.3 mL of 10,000 µg/mL was injected per mouse twice every two days (once each in the morning and evening).

**Figure 8:**
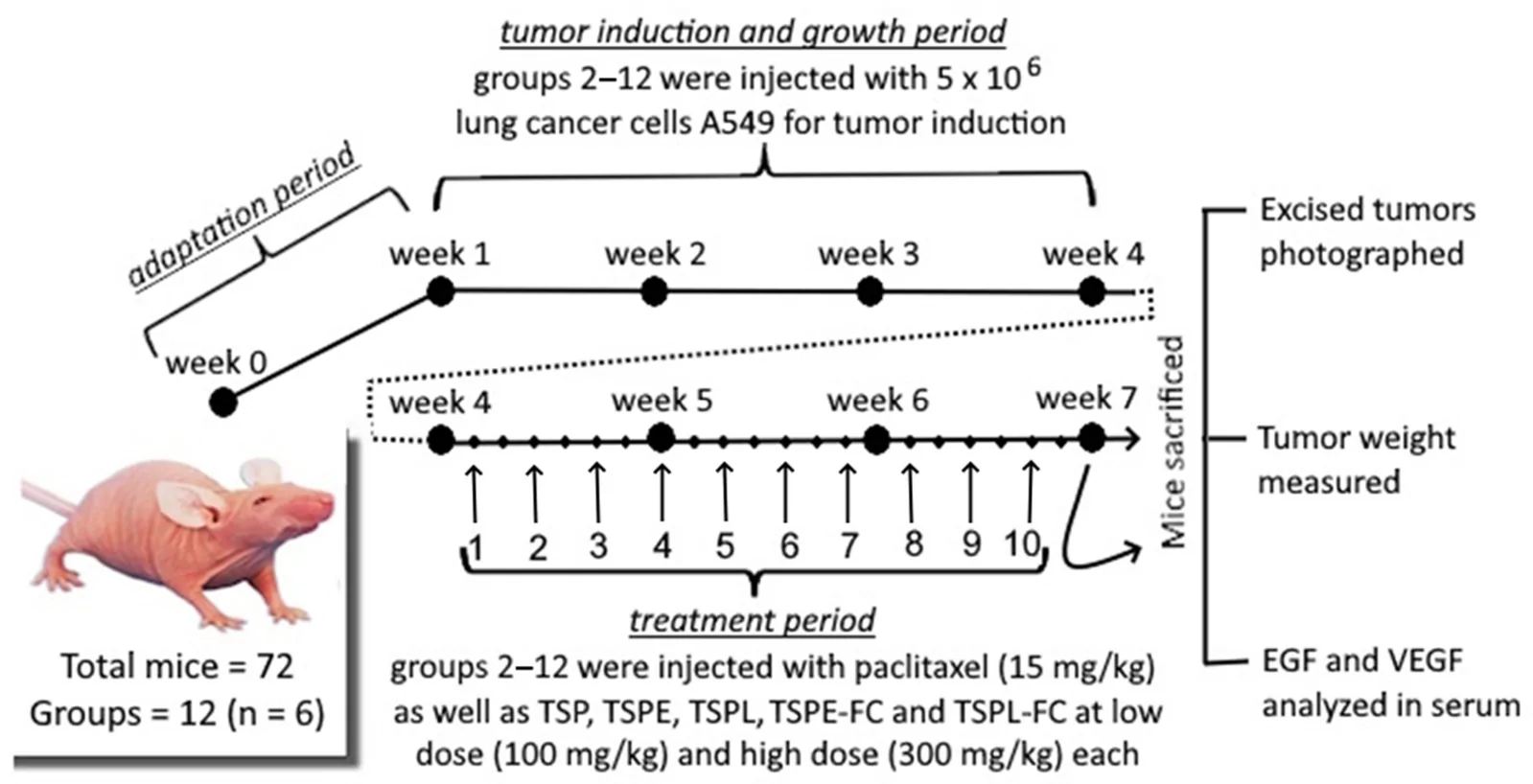
Schematic diagram illustrating the design of animal experiments for studying the antitumor effects of paclitaxel, TSP, TSPE, TSPL, TSPE-FC and TSPL-FC in nude mice for a total period of 7 weeks including an adaptation period of one week, tumor induction and growth period of 3 weeks and treatment period of 3 weeks. The dotted line denotes the connection between the end of tumor growth period (week 1–3) and the beginning of treatment period (week 4–6). The upward arrows with numbers 1–10 at the bottom indicate respectively the day and number of treatments, while the bent arrow represents the day of mice sacrifice after the completion of treatment period. TSP, tilapia skin collagen peptide; TSPE, tilapia skin collagen peptide nanoemulsion; TSPL, tilapia skin collagen peptide nanoliposome; TSPE-FC, folic acid-chitosan conjugated TSPE; TSPL-FC, folic acid-chitosan conjugated TSPL; EGF, epidermal growth factor; VEGF, vascular endothelial growth factor.

At the end of the treatment period, all mice were euthanized with CO_2_ and blood was withdrawn by cardiac puncture for subsequent centrifugation (3500 rpm, 20 min at 4 °C) and collection of serum for determination of epidermal growth factor (EGF) and vascular endothelial growth factors (VEGF) using commercial kits as shown below. Then, the mice body was dissected carefully to remove tumor for subsequent measurement of tumor weight. Figure 8 shows the schematic diagram illustrating the design of animal experiments for studying the antitumor effects of paclitaxel, TSP, TSPE, TSPL, TSPE-FC and TSPL-FC in nude mice for a total period of 7 weeks including an adaptation period of one week, tumor induction and growth period of 3 weeks and treatment period of 3 weeks.

#### 3.6.2. Determination of EGF and VEGF in Mice Serum

EGF in mice serum was determined by using a commercial kit from Abbkine Inc. (Atlanta, GA, USA) with sensitivity at 5 pg/mL. A 50 µL sample of mice serum was added separately into a 96-well plate containing antibodies, followed by adding horseradish peroxidase (HRP)-conjugate reagent (100 µL), covering with a seal plate membrane, gently shaking and incubating for 60 min at 37 °C. After washing each well 5 times with 20× wash solution and drying, 50 µL of chromogen A and 50 µL of chromogen B were sequentially added, shaken gently for mixing and incubated (15 min at 37 °C) for color development. Then, stop solution (50 µL) was added and loaded into an enzyme-linked immunosorbent assay (ELISA) microplate reader (FlexA-200 model, Medclub Scientific Co. Ltd., Taoyuan City, Taiwan) for measurement of absorbance at 450 nm. Following the same procedure, a standard curve was prepared using 5 concentrations (50, 100, 200, 400 and 800 pg/mL) of EGF standards and based on the linear regression equation, the amount of EGF in mice serum was determined [38,39].

For determination of VEGF in mice serum, a commercial kit from Koma Biotech Co. (Seoul, Korea) with sensitivity at 8 pg/mL was used. Briefly, a 100 µL sample of mice serum was added separately into a 96-well plate containing antibodies and covered with a seal plate membrane. After incubation for 2 h, all of the wells were washed with 1× wash buffer 3 times and 100 µL of 1× mouse VEGF antibody was added to all sample wells and incubated for 1 h. Then, washing was repeated 3 times, followed by adding HRP substrate 3,3′,5,5′-tetramethylbenzidine (100 µL) to all wells, incubating for 15 min, adding stop solution (50 µL) to all wells and loading into an ELISA microplate reader for measurement of absorbance at 450 nm. Based on the same procedure described above, a standard curve was prepared with 7 concentrations (1000, 500, 250, 125, 62.5, 31.25 and 15.63 pg/mL) of VEGF standards and, by using the linear regression equation, the amount of VEGF in mice serum was determined [38,39].

### 3.7. Data Analysis

Characterization of TSP, TSPE, TSPL, TSPE-FC and TSPL-FC, including zeta potential determination and AA analysis by HPLC-DAD, were measured in triplicate (*n* = 3) and mean values were reported. Additionally, both particle size and polydispersity index of TSPE and TSPL by DLS were measured in triplicate and mean values reported. On the other hand, animal experiments were designed with each group containing 6 mice (*n* = 6) and their mean values of body weight, tumor volume and tumor weight as well as triplicate measurements (*n* = 3) of EGF and VEGF in mice serum were assessed for significant difference (*p* < 0.05) through ANOVA by Duncan’s multiple range test by using Statistical Analysis Software (SAS, version 6, SAS Institute Inc., Gary, NC, USA).

## 4. Conclusions

Low-MW collagen peptides (TSP, 172-1904 Da) with high proportions of 172 Da peptide fragments was successfully produced from acid-soluble collagen (TSC) isolated from Taiwan tilapia skin, and its characterization revealed a high proportion of hydrophobic AA including Gly, Ala, Pro, Leu, Val, Met, Phe, Ile, Tyr and Cys. Following encapsulation of TSP into nanoemulsion (TSPE) and nanoliposome (TSPL) and conjugation with folic acid-chitosan ligand (TSPE-FC and TSPL-FC), the average particle size, polydispersity index, zeta potential and surface morphology were measured. Compared to nonencapsulated TSP, TSP encapsulated in TSPE, TSPL, TSPE-FC and TSPL-FC showed a sustained release in an in vitro study, implying enhanced stability and sustained delivery of peptide with improved antitumor activity by nanopeptides. Treatment of A549 cell-induced nude mice tumor with paclitaxel, and two doses (100 and 300 mg/kg BW) of TSP, TSPE, TSPL, TSPE-FC and TSPL-FC showed a dose-dependent decrease in tumor volume and weight, with paclitaxel being the most efficient, followed by folic acid-chitosan ligand conjugated nanopeptides, nanopeptides without conjugation and peptides, implying a higher inhibition effect on tumor growth through active and passive targeting by nanopeptides. Furthermore, all nanopeptides decreased the levels of two critical biomarkers involved in tumor progression, EGF and VEGF, in mice serum more effectively than peptides. Thus, the nanoencapsulation of bioactive peptides through conjugation with folic acid-chitosan active-targeting ligand may be a promising strategy for the treatment of patients with lung cancer. More future clinical trials are needed to verify the antitumor effect in mice observed in this study.

## Data Availability

The original contributions presented in this study are included in the article/Supplementary Material. Further inquiries can be directed to the corresponding author.

## Supplementary Materials

The following supporting information can be downloaded at: https://www.mdpi.com/article/10.3390/ijms27177835/s1.

## List of Abbreviations

AA	amino acids
Ang-α-Mel-Res-Lips	angiopep-2 modified α-melittin resveratrol nanoliposome
APA	American physiological association
ASC	acid soluble collagen
AUC	area under the curve
BW	body weight
Der-L	dermaseptin nanoliposome without pH stimulus
Der-pHSL	dermaseptin pH stimulus nanoliposome
DLS	dynamic light scattering
DNA	deoxyribonucleic acid
EDC	N-(3-dimethylamino propyl)-N′-ethylcarbodiimide hydrochloride
EGF	endothelial growth factor
EGFR	epidermal growth factor receptor
ELISA	enzyme-linked immunosorbent assay
EPR	enhanced permeability and retention
FC	folic acid-chitosan
FMOC	9-fluorenylmethyloxycarbonyl
FOLR1	folate receptor 1
FTIR	Fourier transform infrared spectroscopy
G2/M	Gap 2/mitotic
HED	human equivalent dose
HFF-1	human foreskin fibroblast cells
HPLC-DAD	high-performance liquid chromatography-diode array detection
HRP	horseradish peroxidase
IACUC	institutional animal care and use committee
IC_50_	half-maximal inhibitory concentration
ID	internal diameter
IGF-1	insulin-like growth factor 1
IGFBPs	Insulin-like growth factor binding proteins
IgG, IgE	immunoglobulin G, immunoglobulin E
IL-4, IL-6	interleukin-4, interleukin-6
MALDI-TOF-MS	matrix-assisted laser desorption/ionization time-of-flight mass spectrometry
Mel-HAPS-NPs	melittin-loaded and hyaluronic acid-conjugated phospholipid scaffold nanoparticles
MRSD	maximum recommended starting dose
MW	molecular weight
NF-κB	nuclear factor kappa light chain enhancer of activated B
NLRP3	nucleotide-binding oligomerization domain-like receptor pyrin domain containing 3
OH	hydroxyl group
OPA	ortho-phthalaldehyde
PBS	phosphate buffered saline
PI3K/Akt/mTOR	phosphoinositide 3-kinase/protein kinase B/mammalian target of rapamycina
PSC	pepsin soluble collagen
PVDF	polyvinylidene fluoride
Ras/Raf/MAPK/ERK	rat sarcoma/rapidly accelerated fibrosarcoma/mitogen-activated protein kinase/extracellular signal-regulated kinase pathway
SDS-PAGE	sodium dodecyl sulfate-polyacrylamide gel electrophoresis
SEM	scanning electron microscopy
TEM	transmission electron microscopy
TFDA	Taiwan Food and Drug Administration
TGF-β	transforming growth factor β
TSC	tilapia skin collagen
TSP	tilapia skin peptide
TSPE	tilapia skin peptide nanoemulsion
TSPE-FC	folic acid-chitosan conjugated tilapia skin peptide nanoemulsion
TSPL	tilapia skin peptide nanoliposome
TSPL-FC	folic acid-chitosan conjugated tilapia skin peptide nanoliposome
VEGF	vascular endothelial growth factor
VEGFR-2	vascular epidermal growth factor receptor-2
WHO	world health organization
Wnt/β-catenin	wingless-related integration site/beta-catenin
α-Mel-NPs	α-melittin-lipid nanoparticles
