# Supplementary material for: Antitumor Effects of Collagen Peptide Nanoemulsion and Nanoliposome Prepared from Taiwan Tilapia Skin in a Mouse Model
Source: Int J Mol Sci. 2026 Sep 1;27(17):7835. doi: 10.3390/ijms27177835 (PMC13566499; doi:10.3390/ijms27177835)
Supplement: Supplementary file 1 [file ijms-27-07835-s001.zip › ijms-4441447-supplementary.pdf]

Supplementary materials

for

Antitumor Effects of Collagen Peptide Nanoemulsion and Nanoliposome Prepared from Taiwan Tilapia Skin in a Mouse Model

Baskaran Stephen Inbaraj[1], Kuo-Chiang Hsu[2] and Bing-Huei Chen[1,2,*]

[1]Department of Food Science, Fu Jen Catholic University, New Taipei City 242062, Taiwan; sinbaraj@yahoo.com; 138547@mail.fju.edu.tw

[2]Department of Nutrition, China Medical University, Taichung 404328, Taiwan; kchsu@mail.cmu.edu.tw

*Corresponding author's email: 002622@mail.fju.edu.tw

Materials and methods

Pretreatment and extraction of collagen from tilapia skin

A 100-g sample of freeze-dried tilapia skin waste was stirred with 0.5 M NaOH (1:10, w/v) for 48 h and filtered through a cheese cloth. After washing several times with deionized water, the sample was stirred with 0.5 M CH_3COOH (1:15, w/v) for 48 h and filtered, followed by mixing the filtrate with 2.6 M NaCl for protein precipitation (48 h, 4°C), centrifuging (10,000 g, 10 min), dialyzing through a 25 kDa membrane sequentially in 0.1 M CH_3COOH (24 h) and deionized water (48 h), and freeze-drying to obtain the acid-soluble collagen (TSC) with the yield at 23.36 g [1].

Enzymatic hydrolysis of TSC

A 0.5-g TSC sample was suspended in deionized water (1:30, w/v) and heated at 90°C (20 min) for enzyme inactivation. After cooling, the mixture was adjusted to pH 8, heated to 50°C and 12.5% alcalase added for enzymatic hydrolysis with stirring for 6 h. Then the reaction was stopped by heating the mixture (100°C, 20 min), centrifuging (10,000 g, 10 min) and collecting a portion of the supernatant for analysis of degree of hydrolysis, while the remaining supernatant was freeze-dried to obtain tilapia skin collagen peptide (TSP) [1].

Determination of degree of hydrolysis of TSP

Initially, o-phthalaldehyde (OPA) solution was prepared by mixing sodium dodecyl sulfate (20 mg), sodium tetraborate decadhydrate (7.62 g), OPA reagent (160 mg in 4 mL ethanol) and dithiothreitol (176 mg) in deionized water (200 mL). Then serine calibration curve was prepared with 6 concentrations ranging from 0.01-0.10 mg/mL by individually mixing with OPA solution (3 mL), reacting for 2 min (25°C) and measuring absorbance at 340 nm with an U1900 Hitachi Spectrophotometer (Tokyo, Japan). The peptide concentration in TSP (E_{HP}) was determined using the serine standard curve by mixing OPA solution (3 mL) with hydrolyzed TSC sample (0.4 mL) and repeating the same procedure. After measuring the total peptide concentration (A_{HP}) through complete acid (6 N HCl) hydrolysis of freeze-dried TSC and repeating the above procedure, the degree of hydrolysis of TSP in percentage was calculated using the formula: (E_{HP}/A_{HP}) x 100 [2].

Characterization of TSC and TSP

TSC type and TSP MW determination by SDS-PAGE analysis

The TSC type and TSP MW were determined by SDS-PAGE analysis by following the reported methods [1,3]. A 0.1-g sample of TSC or TSP dissolved in water was adjusted to pH 6.8 using Tris-HCl buffer at 1:1 (v/v) ratio, followed by heating for 5 min in a 95°C water bath and centrifuging (14,800 g for 10 min at 4°C). Then, the TSC supernatant (20 μL) was loaded into the polyacrylamide gel sample tank containing 4% stacking gel and 8% separating gel along with loading 10 μL of standard protein marker (MW, 2-250 kDa) separately in the same gel tank. Likewise, in another gel sample tank containing 15% separating gel and 4% stacking gel, 20 μL of TSP supernatant and 10 μL of standard protein marker (MW, 3-260 kDa) were loaded separately. Then, two electrophoretic runs were done individually at 120 V for subsequent staining of gel using Coomassie Brilliant blue solution and destaining in a mixture of methanol, glacial acetic acid and deionized water (40:10:50, v/v/v) until the clear protein bands appeared [3].

Zeta potential analyses

The zeta potential of TSC and TSP samples were determined using Horiba zeta potential analyzer (Kyoto, Japan) by dissolving 1 mg of each sample dissolved in deionized water (2 mL) followed by sonicating (5 min) and pipetting 300 μL into a folded capillary cell and measuring in the potential range of -200 to +200 mV [3].

Scanning electron microscopic analyses

Surface morphology of TSC and TSP was visualized by dusting each sample on a double-sided carbon adhesive tape pasted on a 1.5 cm aluminum stub, removing the excess sample, coating the sample specimen with gold/palladium (MSP-1S, Magnetron sputtering machine (Vacuum Device Inc., Mito, Japan) and mounting on a TM4000 Plus Hitachi Scanning Electron Microscope (SEM; Taipei, Taiwan) for imaging at 15 kV [1].

Preliminary study on determination of antitumor efficiency

An approval was obtained for performing a preliminary study from the Institutional Animal Care and Use Committee (IACUC Approval number A11237, dated April 9, 2024) and an approved guidelines recommended by the American Physiological Association (APA) was followed for ethical conduct in the care and use of animals [4]. Fifty-one 4-week-old male nude mice with each weighing about 20 g were procured from BioLASCO (Taipei, Taiwan) and housed in Fu Jen Catholic University Animal Research Center in ventilated cages and provided with sterilized water and standard laboratory rodent chow ad libitum (Lab Diet 5001). Moreover, a 12-h light/dark cycle was maintained in the animal room with a temperature of 22°C±2°C and relative humidity of 55±10% controlled throughout the experiments.

Initially, human lung cancer cells A549 (ATCC-CCL-185) procured from the Biore-source Collection and Research Center (BCRC), Taiwan Food Industry Research and De-velopment Institute (Hsinchu, Taiwan) were cultured in 90 % Ham's F12K medium with 2 mM L-glutamine adjusted to contain 1.5 g/L sodium bicarbonate and 10 % FBS. After an initial adaptation period of one week for mice, about 5 x 10^6 human lung cancer cells A549 were injected into the mice flank for tumor induction. By using a vernier caliper, the tumor size was measured twice a week and the tumor volume was calculated by substituting length (L, mm) and width (W, mm) of tumor in the formula, $(L \times W^2) / 2$ [5,6]. When the tumor volume attained ~250 mm^3 3 weeks after induction, all 51 mice were divided into 17 groups (n = 3) and administered with the following treatments via intraperitoneal injection for 3 weeks: Group 1 (control) injected with 0.85% saline, Group 2 (positive control) injected with 15 mg/kg body weight (BW) paclitaxel drug, Group 3 (TSP100) injected with a low TSP dose of 100 mg/kg BW, Group 4 (TSP200) injected with a medium TSP dose of 200 mg/kg BW, Group 5 (TSP300) injected with a high TSP dose of 300 mg/kg BW, Group 6 (TSPE100) injected with a low TSPE dose of 100 mg/kg BW, Group 7

(TSPE200) injected with a medium TSPE dose of 200 mg/kg BW, Group 8 (TSPE300) injected with a high TSPE dose of 300 mg/kg BW, Group 9 (TSPL100) injected with a low TSPL dose of 100 mg/kg BW, Group 10 (TSPL200) injected with a medium TSPL dose of 200 mg/kg BW, Group 11 (TSPL300) injected with a high TSPL dose of 300 mg/kg BW, Group 12 (TSPE-FC100) injected with a low TSPE-FC dose of 100 mg/kg BW, Group 13 (TSPE-FC200) injected with a medium TSPE-FC dose of 200 mg/kg BW, Group 14 (TSPE-FC300) injected with a high TSPE-FC dose of 300 mg/kg BW, Group 15 (TSPL-FC100) injected with a low TSPL-FC dose of 100 mg/kg BW, Group 16 (TSPL-FC200) injected with a medium TSPL-FC dose of 200 mg/kg BW and Group 17 (TSPL-FC300) injected with a high TSPL-FC dose of 300 mg/kg BW.

Positive control drug paclitaxel treatment at 15 mg/kg BW dose in Group 2 was conducted by injecting 0.3 mL of 1000 µg/mL paclitaxel solution per mouse. For TSP, TSPE and TSPL treatments, an injection volume of 0.1 mL, 0.2 mL and 0.3 mL of each formulation at 20,000 µg/mL was injected per mouse respectively for low dose treatments (Groups 3, 6, 9), medium dose treatments (Groups 4, 7, 10) and high dose treatments (Groups 5, 8, 11). However, an injection volume of 0.2 mL of 10,000 µg/mL was injected per mouse once every two days for low dose of TSPE-FC treatment in Group 12 and low dose of TSPL-FC treatment in Group 15, while for medium dose of TSPE-FC (Group 13) and TSPL-FC (Group 16) treatments, 0.4 mL of 10,000 µg/mL was injected per mouse once every two days. For high dose treatments of TSPE-FC (Group14) and TSPL-FC (Group 17), 0.3 mL of 10,000 µg/mL was injected per mouse twice every two days (once each in the morning and evening).

References

1 Inbaraj, B.S.; Lai, Y.W.; Chen, B.H. A comparative study on inhibition of lung cancer cells by nanoemulsion, nanoliposome, nanogold and their folic acid conjugates prepared with collagen peptides from Taiwan tilapia skin. *Int. J. Biol. Macromol.* **2024**, *261*, 129722.

2 Hou, N.T.; Chen, B.H. Extraction, purification and characterization of collagen peptide prepared from skin hydrolysate of sturgeon fish. *Food Qual. Saf.* **2023**, *74*, 1-15.

3 Lin, M.Z.; Chen, B.H. An improved production method of bioactive peptides from sturgeon fish cartilage. *Food Bioprocess Technol.* **2025**, *18*, 2050-2069.

4 APA. Guidelines for Ethical Conduct in the Care and Use of Animals, American Physiological Association, 2022. http://www.apa.org/science/leadership/care/guidelines (Accessed on 15 April 2026).

5 Lin, Y.H.; Wang, C.C.; Lin, Y.H.; Chen, B.H. Preparation of catechin nanoemulsion from oolong tea leaf waste and its inhibition of prostate cancer cells DU-145 and tumors in mice. *Molecules* **2021**, *26*, 3260.

6 Hsu, H.Y.; Chen, B.H. A comparative study on inhibition of breast cancer cells and tumors in mice by carotenoid extract and nanoemulsion prepared from sweet potato (*Ipomoea batatas* L.) peel. *Pharmaceutics* **2022**, *14*, 980.

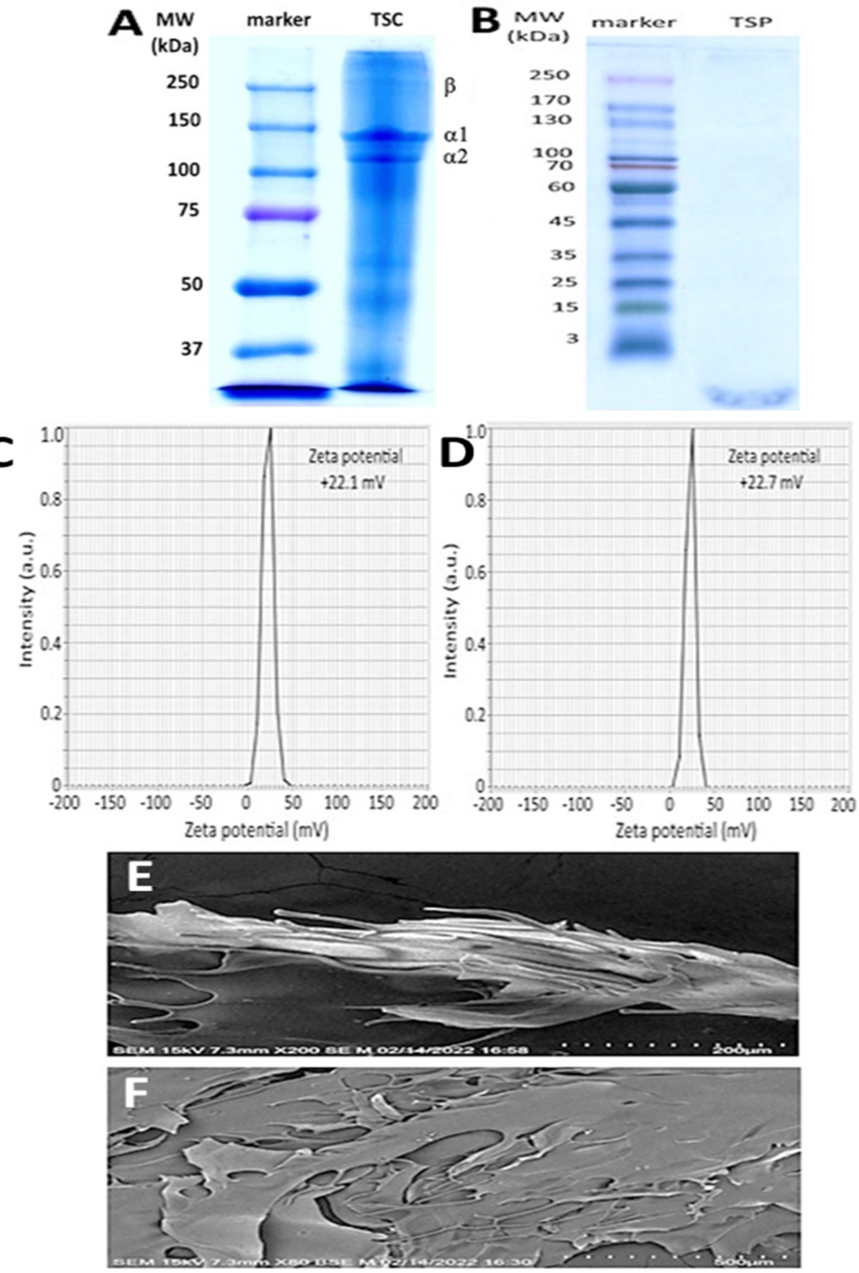

Figure S1. Characterization of TSC and TSP by SDS-PAGE (A, TSC; B, TSP), surface charge by zeta potential analysis (C, TSC; D, TSP) and surface morphology by SEM (E, TSC; F, TSP). Analysis of zeta potential was done in triplicate (n = 3) and the mean values are reported in Figure S1C,D. TSC, tilapia skin acid-soluble collagen; TSP, tilapia skin collagen peptide with MW 172 Da obtained by enzymatic hydrolysis of TSC using 12.5% alcalase; MW, molecular weight; SDS-PAGE, sodium dodecyl sulfate-polyacrylamide gel electrophoresis; SEM, scanning electron microscopy.

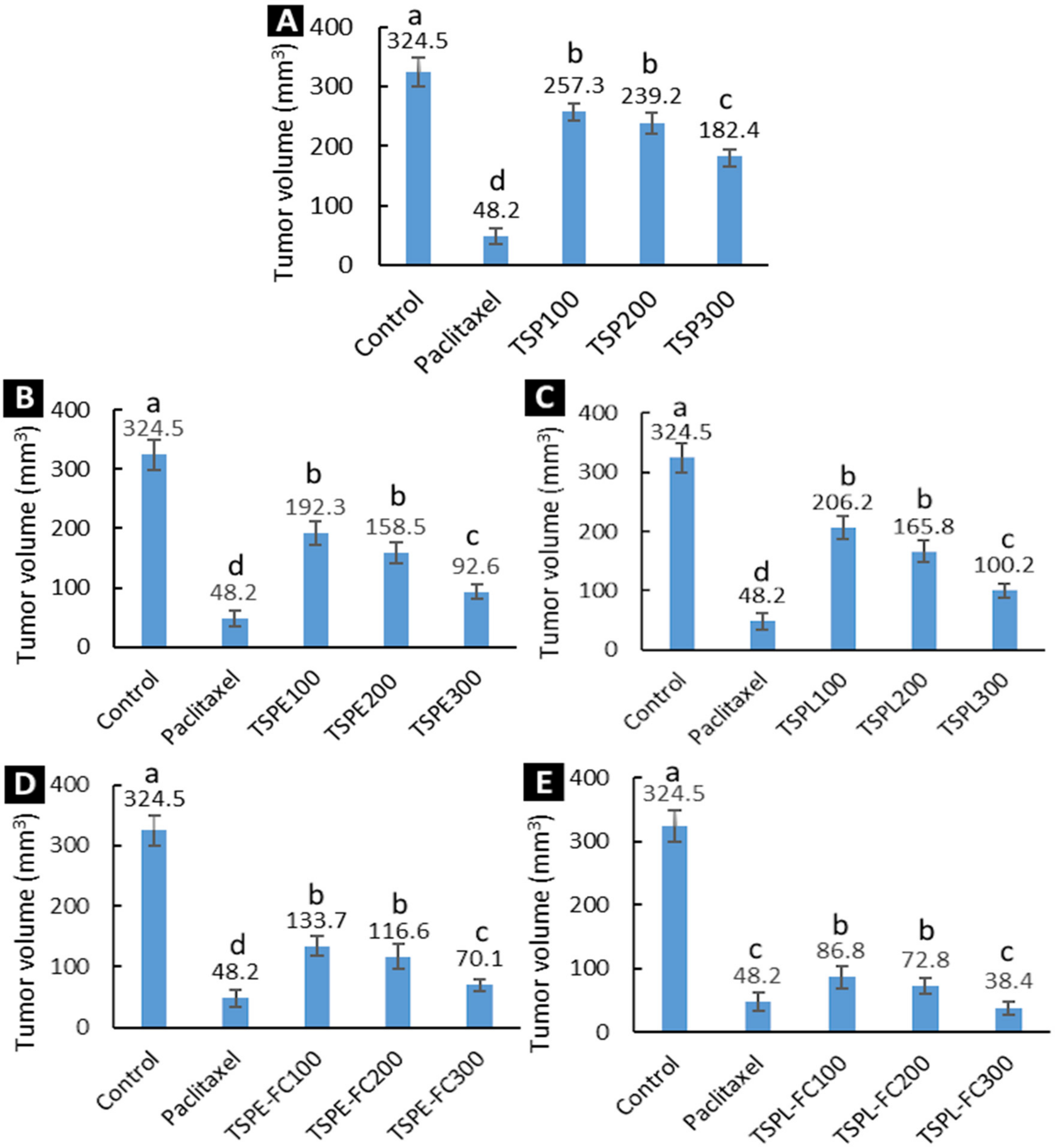

Figure S2. Preliminary study results on the effect of tilapia skin collagen peptides (TSP) (A)and their nanopeptides (TSPE and TSPL) (B,C) as well as folic acid-chitosan ligand conjugated nanopeptides (TSPE-FC and TSPL-FC) (D,E) on tumor size in comparison with control (0.85% saline) and paclitaxel (15 mg/kg BW) in lung tumor-bearing nude mice. TSP, TSPE, TSPL, TSPE-FC and TSPL-FC were injected intraperitoneally with each at three different doses (100, 200 and 300 mg/kg BW) for a 3-week treatment period. Data are presented as mean ± standard deviation of measurements for three nude mice (n = 3) and different small letters (a-d) over the data labels represent significantly different values at $p<0.05$. BW, body weight.

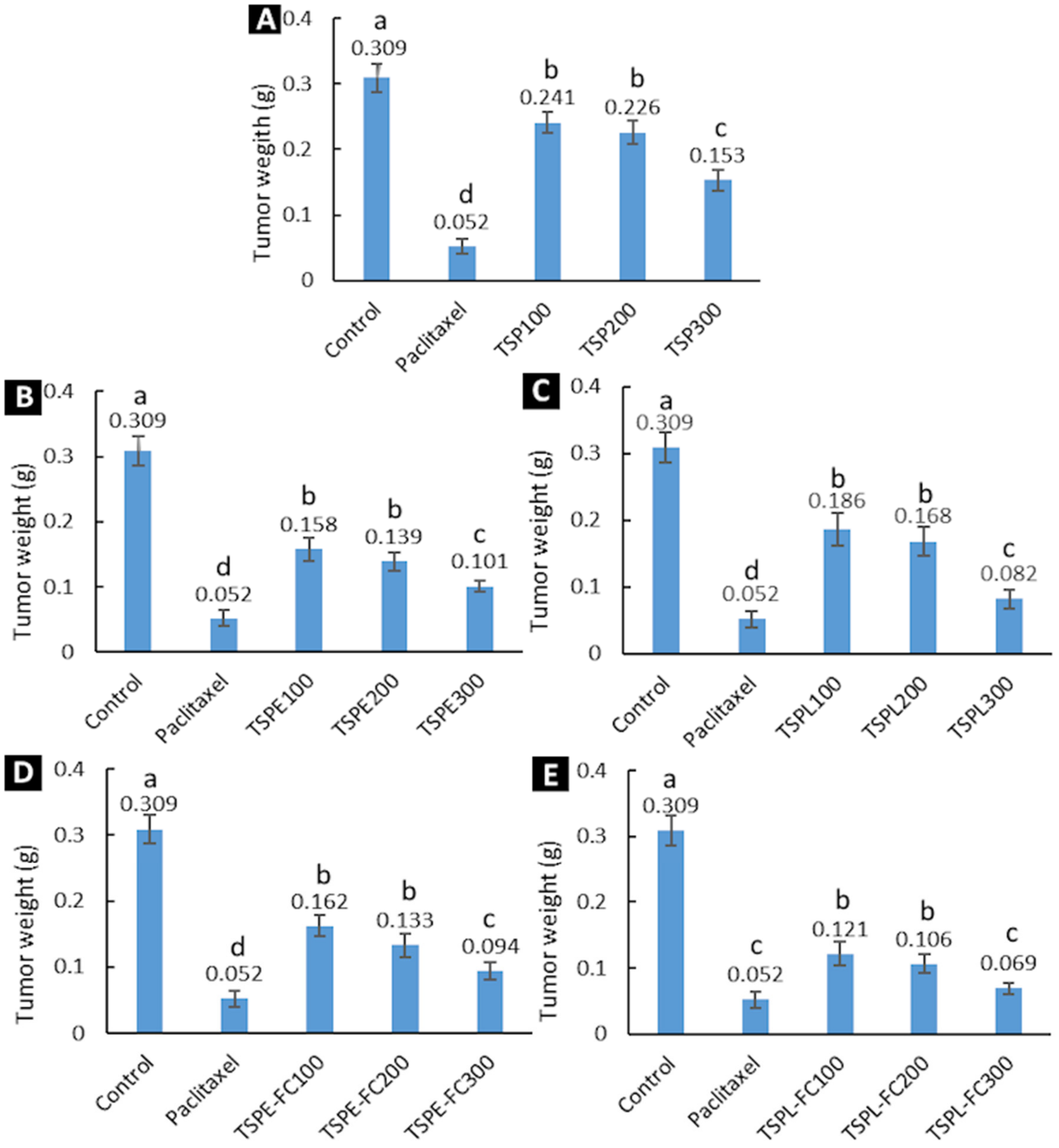

Figure S3. Preliminary study results on the effect of tilapia skin collagen peptides (TSP) (A)and their nanopeptides (TSPE and TSPL) (B,C) as well as folic acid-chitosan ligand conjugated nanopeptides (TSPE-FC and TSPL-FC) (D,E) on tumor weight in comparison with control (0.85% saline) and paclitaxel (15 mg/kg BW) in lung tumor-bearing nude mice. TSP, TSPE, TSPL, TSPE-FC and TSPL-FC were injected intraperitoneally with each at three different doses (100, 200 and 300 mg/kg BW) for a 3-week treatment period. Data are presented as mean ± standard deviation of measurements for three nude mice (n = 3) and different small letters (a-d) over the data labels represent significantly different values at $p<0.05$. BW, body weight.

Table S1. Amino acid composition of TSP prepared from TSC isolated from tilapia skin.

Amino acid	TSP (Number of residues/1000 residues[1]
Alanine	135.98 ± 0.82
Arginine	54.64 ± 0.52
Aspartic acid	48.40 ± 0.59
Cysteine	2.81 ± 0.12
Glutamic acid	65.30 ± 0.68
Glycine	331.29 ± 1.18
Histidine	12.20 ± 0.32
Hydroxyproline	73.44 ± 0.86
Isoleucine	13.24 ± 0.34
Leucine	26.27 ± 0.40
Lysine	28.47 ± 0.63
Methionine	19.16 ± 0.80
Phenylalanine	16.97 ± 0.91
Proline	100.40 ± 1.20
Serine	21.02 ± 0.90
Threonine	23.33 ± 0.49
Tyrosine	6.20 ± 0.27
Valine	20.88 ± 0.32
Total	1000

[1]Data shown are mean ± standard deviation of triplicate experiments. TSC, acid soluble collagen isolated from tilapia skin; TSP, tilapia skin collagen peptide with MW 172 Da prepared by enzymatic hydrolysis of TSP using 12.5% alcalase; MW, molecular weight.
